# Supplementary material for: Methodological Flaws in Meta-Analyses of Clinical Studies on the Management of Knee Osteoarthritis with Stem Cells: A Systematic Review
Source: Cells. 2022 Mar 11;11(6):965. doi: 10.3390/cells11060965 (PMC8946093; doi:10.3390/cells11060965)
Supplement: Supplementary file 1 [file cells-11-00965-s001.zip › cells-1622920-supplementary.pdf]

Methodological flaws in meta-analyses of clinical studies on treatment of knee osteoarthritis with stem cells

Christoph Schmitz, Christopher Alt, David Pearce,

John P. Furia, Nicola Maffulli and Eckhard U. Alt

Supplementary Material

Table S1 | Details of studies that were identified during an evidence-based, systematic review of the literature according to the PRISMA guidelines [198] performed on August 07, 2021 in order to examine studies on treatment of primary knee osteoarthritis with stem cells.

Generell abbreviations: R, reference; C, category of study as defined in Table 1 in the main text; T, type of study as defined in Table 2 in the main text; FA, first author; Y, year of publication; Ce, cell type; O, origin (autologous or allogeneic); allo, allogeneic; auto, autologous; N, number of patients in the treatment group; Cu, culturing of cells.

Abbreviations of cell types: ADRCs, adipose-derived regenerative cells; ADSCs, adipose-derived stem cells (obtained by culturing ADRCs); BMA, bone marrow aspirate; BMAC, bone marrow aspirate concentrate; BM-MSCs, bone marrow-derived mesenchymal stem cells; Ch-TGFß, chondrocytes overexpressing transforming growth factor beta; CLL; centrifuged liposuction liquid; Cs, chondrocytes; CSCs, cartilage stem cells; hUC-MSCs, human umbilical cord-derived MSCs; hUCB-MSCs, human umbilical cord blood-derived MSCs; IPFP-SCs, stem cells isolated from human infrapatellar fat pad; MACI; matrix-induced autologous chondrocyte implant; MFF, micro-fragmented fat (from liposuction); pBSCs, activated peripheral blood stem cells; P-MSCs, placental MSCs; S-MSCs, matrix-induced MSCs from synovia.

Abbreviations of treatments: AD, arthroscopic debridement; AlCh, allogeneic cartilage; AM, athroscopic repair of meniscus; AMIC, autologous matrix-induced chondrogenesis; AP; autologous periosteum; AuCh, autologous chondrocytes; BMAC; bone marrow aspirate concentrate; Ccs, subchondral application of cells; CF, centrifuged fat; CM, conservative management; CS, corticosteroid; DFO, distal femoral osteotomy; FF, filtrated fat; GF, growth factor; HA, hyluronic acid; HTO, high tibial osteotomy; MACI, matrix-induced, autologous chondrocyte implant; MF, microfracture; OA, oral acetaminophen; PL, platelet lysate; PRP, platelet rich plasma; RS, Ringer solution; Sa, saline; S, scaffold; SR, surgical repair; TPA, total knee arthroplasty (internal control);

Remark: a, in one study [120] a combination of autologous BMAC + PRP + PL was administered into the knee joint and the subchondral bone (treatment group), or into the knee joint alone (control group), respectively; b, this study [238] was published after we had performed our evidence-based, systematic review of the literature on August 07, 2021. Because it is a first-in-human study applying stem cells from a new source (infrapatellar fat pad) this study [238] was added to this table.

R	C	T	FA	Y	Ce	O	Treatment	N	Control	Cu
[12]	I	a	Cherian	2015	Ch-TGFß	allo	C	50	Sa	Yes
[13]	I	a	Gupta	2016	BM-MSCs	allo	C + HA	40	C or S	Yes
[14]	I	a	Emadedin	2018	BM-MSCs	auto	C	19	Sa	Yes
[15]	I	a	Kim	2018	Ch-TGFß	allo	C	78	Sa	Yes
[16]	I	a	Kuah	2018	ADSCs	allo	C	16	Sa	Yes
[17]	I	a	Khalifeh Soltani	2019	P-MSCs	allo	C	10	Sa	Yes
[18]	I	a	Lee	2019	ADSCs	auto	C	12	Sa	Yes
[19]	I	a	Garza	2020	ADRCs	auto	C	26	RS	No
[20]	II	a	Vega	2015	BM-MSCs	allo	C	15	HA	Yes
[21]	II	a	Wang	2016	hUC-MSCs	allo	C	18	HA	Yes
[22]	II	a	Goncars	2017	BMAC	auto	C	28	HA	No
[23]	II	a	Lu	2019	ADSCs	auto	C	26	HA	Yes
[24]	II	a	Matas	2019	hUC-MSCs	allo	C	18	HA	Yes
[25]	II	a	Anz	2020	BMAC	auto	C	45	PRP	No
[26]	II	a	Dallo	2021	MFF	auto	C	40	PRP+HA	No
[27]	II	d	Kim	2020	ADRCs	auto	C	30	HA	No

Table S1 (cont.)

R	C	T	FA	Y	Ce	O	Treatment	N	Control	Cu
[28]	III	a	Garay-Mendoza	2018	BMAC	auto	C	61	OA	No
[29]	III	a	Song	2018	ADSCs	auto	C	18	C	Yes
[30]	III	a	Freitag	2019	ADSCs	auto	C	20	CM	Yes
[31]	III	a	Zhao	2019	ADSCs	allo	C	18	C	Yes
[32]	III	a	Bastos	2020	BM-MSCs	auto	C	16	C + PRP +CS	Yes
[33]	III	a	Lu	2020	ADSCs	allo	C	22	C	Yes
[34]	III	b	Hernigou	2018	BMAC	auto	C	30	TPA	No
[35]	III	b	Hernigou	2020	BMAC	auto	C	60	Csc	No
[36]	III	b	Hernigou	2020	BMAC	auto	C	140	TPA	No
[37]	III	c	Jo	2014	ADSCs	auto	C	18	C	Yes
[38]	III	c	Pers	2016	ADSCs	auto	C	18	C	Yes
[39]	III	c	Jo	2017	ADSCs	auto	C	18	C	Yes
[40]	III	c	Pers	2018	ADSCs	auto	C	18	C	Yes
[41]	III	c	Chahal	2019	ADSCs	auto	C	12	C	Yes
[42]	III	c	Estrada	2020	ADRCs	auto	C	33	BMAC + PRP	No
[43]	III	c	Kazemian	2020	BM-MSCs	auto	C	20	Not described	Yes
[44]	III	c	Papalia	2020	MFF	auto	C	8	CF or FF	No
[45]	III	c	Bistolfi	2021	MFF	auto	C	27	CF	No
[46]	III	d	Mautner	2019	MFF	auto	C	23	BMAC	No
[47]	III	d	Yokota	2019	ADRCs	auto	C	38	C	No
[48]	III	d	Li	2020	BM-MSCs	auto	C	40	AD + HA	Yes
[49]	III	d	Simunec	2020	ADRCs	auto	C	6	C + PRP	No
[50]	IV	a	Varma	2010	BM-MSCs	auto	C + AD	25	AD	Yes
[51]	IV	a	Wong	2013	BM-MSCs	auto	C + MF + HTO + HA	28	HA + HTO + MF	Yes
[52]	IV	a	Koh	2014	ADRCs	auto	C + AD + HTO + PRP	21	PRP + AD + HTO	No
[53]	IV	a	Lamo-Espinosa	2016	BM-MSCs	auto	C + HA	10	HA	Yes
[54]	IV	a	Turajane	2017	pBSCs	auto	C + AD + MF	20	HA	No
[55]	IV	a	Bastos	2018	BM-MSCs	auto	C + PRP	9	C	Yes
[56]	IV	a	Zhang	2018	ADSCs	auto	C + HA	36	C	Yes
[57]	IV	a	Lamo-Espinosa	2018	BM-MSCs	auto	C + HA	10	HA	Yes
[58]	IV	a	Peretti	2018	MFF	auto	C + AD	20	AD	No
[59]	IV	a	Lamo-Espinosa	2020	BM-MSCs	auto	C + PRP	30	PRP	Yes
[60]	IV	a	Qiao	2020	ADSCs	auto	C + AD + MF + HA	10	MF or MF + HA	Yes
[61]	IV	a	Ruane	2021	BMAC	auto	C + PRP	17	HA	No
[62]	IV	b	Shapiro	2017	BMAC	auto	C + PPP	25	S	No
[63]	IV	b	Hong	2019	ADRCs	auto	C + AD	16	HA + AD	No
[64]	IV	b	Shapiro	2019	BMAC	auto	C + PPP	25	S	No

Table S1 (cont.)

R	C	T	FA	Y	Ce	O	Treatment	N	Control	Cu
[65]	IV	c	Koh	2012	ADSCs	auto	C + AD + PRP	25	AD + PRP	?
[66]	IV	c	Kim	2015	ADRCs	auto	C + AD + PRP	20	C in S + AD	No
[67]	IV	c	Nguyen	2017	ADRCs	auto	C + AD + MF + PRP	15	AD + MF	No
[68]	IV	c	Kim	2018	ADRCs	auto	C + AD + HTO	50	HTO	No
[69]	IV	c	Tran	2019	ADRCs	auto	C + AD + MF	18	AD + MF	No
[70]	IV	c	Ehlers	2020	ADRCs	auto	C + PRP	8	PRP	No
[71]	IV	d	Centeno	2014	BMAC	auto	C + adipose graft	840	C	No
[72]	IV	d	Kim	2015	ADRCs	auto	C in S + AD	17	C + AD	No
[73]	IV	d	Srinivas	2015	BMAC	auto	C + PRP +Co	65	Co	No
[74]	IV	d	Lee	2021	BMAC	auto	C + MF + HTO	74	hUCB-MSCs	No
[75]	IV	d	Centeno	2021	BMAC	auto	BMAC + PRP + PL[a]	80	BMAC + PRP +PL[a]	
[76]	IV	d	Magnanelli	2021	MFF	auto	C + HTO	42	HTO	No
[77]	V	e	Davatchi	2011	BM-MSCs	auto	C	4	None	Yes
[78]	V	e	Pak	2011	ADRCs	auto	C + PRP + HA	2	None	No
[79]	V	e	Emadedin	2012	BM-MSCs	auto	C	6	None	Yes
[80]	V	e	Hauser	2013	BMA	auto	C	3	None	No
[81]	V	e	Koh	2013	ADRCs	auto	C + PRP + HA	33	None	No
[82]	V	e	Orozco	2013	BM-MSCs	auto	C	12	None	Yes
[83]	V	e	Turajane	2013	pBSCs	auto	C + MF + GF + HA	4	None	No
[84]	V	e	Ahmad	2014	pBSCs	auto	C	10	None	No
[85]	V	e	Bui	2014	ADRCs	auto	C + PRP	23	None	No
[86]	V	e	Koh	2014	ADRCs	auto	C + AD	37	None	No
[87]	V	e	Orozco	2014	BM-MSCs	auto	C	12	None	Yes
[88]	V	e	Centeno	2015	BMAC	auto	C	373	None	No
[89]	V	e	Emadedin	2015	BM-MSCs	auto	C	6	None	Yes
[90]	V	e	Gibbs	2015	ADRCs	auto	C + PRP	4	None	No
[91]	V	e	Kim	2015	ADRCs	auto	C in S + AD	55	None	No
[92]	V	e	Koh	2015	ADRCs	auto	C +AL	30	None	No
[93]	V	e	Davatchi	2016	BM-MSCs	auto	C	4	None	Yes
[94]	V	e	Fodor	2016	ADRCs	auto	C	6	None	No
[95]	V	e	Kim	2016	ADRCs	auto	C in S + AD	20	None	No
[96]	V	e	Pak	2016	ADRCs	auto	C + PRP + HA	3	None	No
[97]	V	e	Sampson	2016	BMAC	auto	C + PRP	125	None	No
[98]	V	e	Soler	2016	BM-MSCs	auto	C	15	None	Yes
[99]	V	e	Al-Najar	2017	BM-MSCs	auto	C	13	None	Yes
[100]	V	e	Hudetz	2017	MFF	auto	C	17	None	No
[101]	V	e	Park	2017	hUCB-MSCs	allo	C + MF	6	None	Yes
[102]	V	e	Pintat	2017	ADRCs	auto	C + PRP	19	None	?

Table S1 (cont.)

R	C	T	FA	Y	Ce	O	Treatment	N	Control	Cu
[103]	V	e	Russo	2017	MFF	auto	C	30	None	No
[104]	V	e	Smyshlyaev	2017	ADRCs	auto	C	28	None	No
[105]	V	e	Yokota	2017	ADRCs	auto	C	13	None	No
[106]	V	e	Cattaneo	2018	MFF	auto	C	38	None	No
[107]	V	e	Cavallo	2018	BMAC	auto	C + AD + PRP	24	None	No
[108]	V	e	Rodriguez-Fontan	2018	BMAC	auto	C	10	None	No
[109]	V	e	Russo	2018	MFF	auto	C	30	None	No
[110]	V	e	Shaw	2018	BMAC	auto	C	15	None	No
[111]	V	e	Spasovski	2018	ADSCs	auto	C	9	None	Yes
[112]	V	e	Themistocleous	2018	BMAC	auto	C	233	None	No
[113]	V	e	Borić	2019	MFF	auto	C	17	None	No
[114]	V	e	Goncars	2019	BMAC	auto	C	32	None	No
[115]	V	e	Hudetz	2019	MFF	auto	C	20	None	No
[116]	V	e	Monckeberg	2019	pBSCs	auto	C + PRP	20	None	Yes
[117]	V	e	Onoi	2019	ADRCs	auto	C	2	None	No
[118]	V	e	Roato	2019	CLL	auto	C + AD	20	None	No
[119]	V	e	Schiavone Panni	2019	MFF	auto	C + AD	52	None	No
[120]	V	e	Song	2019	hUCB-MSCs	allo	C + DFO	2	None	Yes
[121]	V	e	Wang	2019	BM-MSCs	auto	C + chondrocytes	2	None	Yes
[122]	V	e	Colberg	2020	BMAC	auto	C	10	None	No
[123]	V	e	Dilogo	2020	hUC-MSCs	allo	C	39	None	Yes
[124]	V	e	Dulic	2020	BMAC	auto	C	111	None	No
[125]	V	e	Freitag	2020	ADSCs	auto	C + AD	27	None	Yes
[126]	V	e	Heidari	2020	MFF	auto	C	110	None	No
[127]	V	e	Higuchi	2020	ADSCs	auto	C	34	None	Yes
[128]	V	e	Kim	2020	BMAC	auto	C	25	None	No
[129]	V	e	Kim	2020	ADRCs	auto	C in S	467	None	No
[130]	V	e	Lapuente	2020	ADRCs	auto	C	50	None	No
[131]	V	e	Mehling	2020	ADRCs	auto	C + PRP	241	None	No
[132]	V	e	Prodromos	2020	MFF	auto	C + PRP	42	None	No
[133]	V	e	Song	2020	hUCB-MSCs	allo	C + AD + MF + HA	128	None	Yes
[134]	V	e	Song	2020	hUCB-MSCs	allo	C + MF + HTO + HA	25	None	Yes
[135]	V	e	Song	2020	hUCB-MSCs	allo	C + MF + HTO + HA	25	None	Yes
[136]	V	e	Toan	2020	BM-MSCs	auto	C + AD	46	None	?
[137]	V	e	Tsubosaka	2020	ADRCs	auto	C	57	None	No
[138]	V	e	Varady	2020	BMAC	auto	C	17	None	No
[139]	V	e	Wells	2020	BMAC	auto	C	11	None	No
[140]	V	e	Bakowski	2021	MFF	auto	C	59	None	No

Table S1 (cont.)

R	C	T	FA	Y	Ce	O	Treatment	N	Control	Cu
[141]	V	e	Borg	2021	MFF	auto	C	456	None	No
[142]	V	e	Burnham	2021	BMAC	auto	C	112	None	No
[143]	V	e	Caforio	2021	MFF	auto	C + AD	30	None	No
[144]	V	e	Heidari	2021	MFF	auto	C	344	None	No
[145]	V	e	Kim	2021	ADRCs	auto	C + AD + HTO	75	None	No
[146]	V	e	Malanga	2021	MFF	auto	C	20	None	No
[147]	V	e	Santoprete	2021	ADRCs	auto	C	84	None	No
[148]	V	e	Sekiya	2021	S-MSCs	auto	C	8	None	Yes
[149]	V	e	Van Genechten	2021	MFF	auto	C	64	None	No
[150]	V	f	Centeno	2008	BM-MSCs	auto	C	1	None	Yes
[151]	V	f	Centeno	2008	BM-MSCs	auto	C	1	None	Yes
[152]	V	f	Mehrabani	2016	BM-MSCs	auto	C	1	None	Yes
[153]	V	f	Bright	2018	ADRCs	auto	C	1	None	No
[154]	V	f	Freitag	2019	ADSCs	auto	C + AD + HTO	1	None	Yes
[155]	VI	a	Wakitani	2002	BM-MSCs	auto	C + AD + HTO + aP	12	HTO	Yes
[156]	VI	a	Saw	2013	pBSCs	auto	C + MF + HA	25	HA	No
[157]	VI	a	Akgun	2015	S-MSCs	auto	C in S + AD	7	MACI	Yes
[158]	IV	a	Koh	2016	ADRCs	auto	C in S + MF	20	MF	No
[159]	VI	a	Shadmanfar	2018	BM-MSCs	auto	C	15	Placebo	Yes
[160]	VI	a	de Girolamo	2019	BMAC	auto	C + AMIC	12	AMIC	No
[161]	VI	a	Hashimoto	2019	BM-MSCs	auto	C + MF	7	MF	Yes
[162]	VI	a	Olivos-Meza	2019	pBSCs	auto	C in S	17	Sa	Yes
[163]	VI	a	Kim	2020	ADRCs	auto	C + AD + HTO	40	C + AD + HTO + Aca	No
[164]	VI	a	Lin	2021	hUCB-MSCs	allo	C + AD + MF	73	MF	Yes
[165]	VI	a	Saw	2021	pBSCs	auto	C + MF + HA	35	HA	No
[166]	VI	c	Gobbi	2015	BMAC	auto	C in S + AD	19	MACI	No
[167]	VI	c	Gobbi	2016	BMAC	auto	C in S	27	MF	No
[168]	VI	c	Martinčič	2020	BMAC	auto	C in S	9	autoCh	No
[169]	VI	d	Ryu	2020	BMAC	auto	C + AD	52	hUC-MSCs	No
[170]	VI	e	Kasemkijwattana	2011	BM-MSCs	auto	C in S	2	None	Yes
[171]	VI	e	Skowroński	2012	pBSCs	auto	C in S + AD	52	None	No
[172]	VI	e	Buda	2013	BMAC	auto	C in S + AD	20	None	No
[173]	VI	e	Gobbi	2014	BMAC	auto	C in S + AD	25	None	No
[174]	VI	e	Zhang	2014	MACI	auto	C in S + AD	15	None	Yes
[175]	VI	e	Jiang	2016	CSCs	auto	C in S + AD	15	None	Yes
[176]	VI	e	Sadlik	2017	hUC-MSCs	allo	C in S	?	None	Yes
[177]	VI	e	Whitehouse	2017	BM-MSCs	auto	C in S	5	None	Yes
[178]	VI	e	Kamei	2018	BM-MSCs	auto	C	5	None	Yes

Table S1 (cont.)

R	C	T	FA	Y	Ce	O	Treatment	N	Control	Cu
[179]	VI	e	Shetty	2018	BMAC	auto	C + AD + MF	60	None	No
[180]	VI	e	Shimomura	2018	S-MSCs	auto	C	5	None	Yes
[181]	VI	e	Gobbi	2019	BMAC	auto	C in S + AD	23	None	No
[182]	VI	e	Sekiya	2019	S-MSCs	auto	C + SR	6	None	Yes
[183]	VI	e	Ciemniewska-Gorzela	2020	BMAC	auto	C in S + AM	54	None	No
[184]	VI	e	Freitag	2020	ADSCs	auto	C + AD	8	None	Yes
[185]	VI	e	Veber	2020	BMAC	auto	C in S	15	None	No
[186]	VI	e	Chung	2021	hUCB-MSCs	allo	C + AD + HTO	93	None	Yes
[187]	VI	e	Liu	2021	Cs/CPs	auto	C in S + AD	12	None	Yes
[188]	VI	e	Saris	2021	BM-MSCs	allo	C + AD	35	None	Yes
[189]	VI	f	Adachi	2005	BM-MSCs	auto	C in S	1	None	Yes
[190]	VI	f	Kuroda	2007	BM-MSCs	auto	C in S	1	None	Yes
[191]	VI	f	Broyles	2017	BMAC	auto	C + PRP + HA	1	None	No
[192]	VI	f	Freitag	2017	ADSCs	auto	C	1	None	Yes
[193]	VI	f	Freitag	2017	ADSCs	auto	C	1	None	Yes
[194]	VI	f	Leigheb	2017	Cs	auto	C in S	1	None	Yes
[195][b]	V	e	Chen	2022	IPFP-SCs	auto	C	12	None	Yes

Table S2 | Conclusions of 19 meta-analyses (published between January 2020 and July 2021) of studies in which treatment of primary knee osteoarthritis with different types of stem cells were investigated.

Abbreviations: AD = adipose-derived; BM = bone marrow-derived; BMAC = bone marrow aspirate concentrate; HA = hyaluronic acid; KOOS = Knee Injury and Osteoarthritis Outcome Score; MSCs = mesenchymal stem cells; OA = osteoarthritis; PRP = platelet rich plasma; R = reference number (note that the reference numbers provided in this table refer to the reference numbers in the main text); SVF = stromal vascular fraction; WOMAC = Western Ontario and McMaster Universities Osteoarthritis Index.

R	Conclusion (original quotes taken from the abstracts of the cited papers)
**Meta-analyses that demonstrated efficacy of treating pkOA with stem cells**	
[199]	"There are significant therapeutic effects on joint function, symptoms, and no permanent adverse effect has been found after stem cell treatment. It is promising to apply intro-articular injection of stem cells for OA to clinical application."
[200]	"Based on the current studies, our results suggested that MSCs were a promising option for the treatment of patients with knee OA."
[201]	"Intra-articular injection of MSCs is effective and safe to relieve pain and improve motor function of patients with knee OA in a short term which is different to conclusions of previous study."
[202]	"We demonstrated that MSC treatment could significantly decrease visual analog scale in a 12-month follow-up study compared with controls ($p < 0.001$). MSC therapy also showed significant decreases in WOMAC scores after the 6-month follow-up ($p < 0.001$). MSC therapy showed no difference compared with controls ($p > 0.05$) in adverse events. We suggest that MSC therapy could serve as an effective and safe therapy for clinical application in OA treatment."
[203]	"Stem cell therapy is certainly superior to traditional treatments in the conservative treatment of knee OA; it considerably reduces pain with no obvious additional side effects."
[204]	" MSCs relieve pain, stiffness, and dysfunction due to OA better than PRP, HA, and GCs and are not statistically correlated with greater safety concerns
**Meta-analyses that demonstrated superiority of autologous, adipose-derived stem cells over other types of stem cells in treatment of knee OA**	
[205]	"These findings suggested that MSCs are effective in the treatment of knee OA. AD-MSCs might be the most effective for relieving pain, and umbilical cord-derived mesenchymal stem cells might be the most effective for improving function. However, the current evidence does not support the use of MSCs for improving cartilage repair in knee OA patients."
[206]	"Overall, MSC-based cell therapy is a relatively safe treatment that holds great potential for OA, evidenced by a positive effect on pain and knee function. Using low-dose (25 million) and adipose-derived stem cells is likely to achieve better results."
[207]	"A single BMAC or SVF injection into the knee joint of patients with OA resulted in symptomatic improvement at short-term follow-up. However, SVF seemed to be more effective than did BMAC in the reduction of knee pain."
[208]	"Our analysis establishes the efficacy, safety, and superiority of AD-MSC transplantation, compared to BM-MSC, in the management of osteoarthritis of knee from available literature."
[209]	"The therapeutic effect of AD-MSCs on knee OA was more effective than that of BM-MSCs."

Table S2 (cont.)

R	Conclusion
	**Meta-analysis that demonstrated efficacy of treating pkOA with autologous, adipose-derived stem cells, without comparison with other types of stem cells**
[210]	"Pooled analysis revealed that cell-based treatments definitively improve WOMAC scores, post treatment. These improvements increased with time. The studies in this meta-analysis have established the safety and efficacy of both AD-MSC therapy and SVF therapy for knee OA in old adults and show that they reduce pain and improve knee function in symptomatic knee OA suggesting that they may be effective therapies to improve mobility in an aging population."
[211]	"During 6 months of follow-up, AD-MSCs relieved pain the best; LP-PRP was most effective for functional improvement. During the 12-month follow-up, both AD-MSCs and LP-PRP showed potential clinical pain relief effects; functional improvement was achieved with LP-PRP."
	**Meta-analysis that demonstrated superiority of autologous, bone marrow-derived stem cells over autologous, adipose-derived stem cells in treatment of pkOA**
[212]	"Intra-articular injections of MSCs without any adjuvant therapies improves pain and function for osteoarthritis. Significantly better outcomes were obtained with the use of bone marrow MSCs as compared with adipose MSCs and with the use of cultured MSCs as opposed to uncultured MSCs."
	**Meta-analysis that demonstrated efficacy of treating pkOA with autologous, bone marrow-derived stem cells, without comparison with other types of stem cells**
[213]	"Intra-articular injection of culture-expanded MSCs without adjuvant surgery can improve pain for patients experiencing knee osteoarthritis at short-term follow-up (6-12 months)."
	**Meta-analysis that demonstrated efficacy of treating pkOA with allogeneic stem cells**
[214]	"Cell-based therapy had a better effect on KOOS improvement and pain relief without safety concerns. However, cell-based therapy did not show a benefit in terms of the WOMAC. Allogeneic cells might have advantages compared to controls in the WOMAC and KOOS scores."
	**Meta-analysis that demonstrated efficacy of treating pkOA with stem cells only in conjunction with surgery**
[215]	"The pooled standardized mean difference from meta-analyses showed statistically significant effects of MSC on self-reported physical function but not self-reported pain. MSCs provided functional benefit only in patients who underwent concomitant surgery."
	**Meta-analysis that demonstrated lack of efficacy of treating pkOA with stem cells**
[216]	"The ranking statistics like surface under the cumulative ranking curve values of our network meta-analysis support the use of steroids and HA for appropriate patients with knee OA. For pain relief and adverse events, steroids are most likely the best treatment, followed by HA. Single PRP, multiple PRP, and adipose MSC interventions do not result in a relevant reduction of joint pain nor improvement of joint function compared with placebo."
[217]	"Intra-articular MSC injection was not found to be superior to placebo in pain relief and functional improvement for patients with symptomatic knee OA."

Table S3 | Details of the studies included in the meta-analyses summarized in Table 1 in the main text in which treatment of primary knee osteoarthritis (pkOA) with different types of stem cells was investigated.

General abbreviations: R, reference number; C, category of study as shown in Table 2 in the main text; T, Type of study as shown in Table 3 in the main text; ●, study included in the corresponding meta-analysis; N, number of meta-analyses in which the corresponding study was included.

Abbreviations of cell types (in alphabetical order): x ADRCs, autologous, adipose-derived regenerative cells; x ADSCs, autologous, adipose-derived stem cells; y ADSCs, allogeneic, adipose-derived stem cells; x BMACBMAC, autologous bone marrow aspirate concentrate; x BM-MSCs, autologous, bone marrow-derived mesenchymal stem cells; y BM-MSCs, allogeneic, bone marrow-derived mesenchymal stromal cells; y Ch-TGFß, allogeneic chondrocytes that overexpress transcription growth factor beta; x CLL, autologous, centrifuged liposuction liquid; y hUC-MSCs, allogeneic, human umbilical cord-derived MSCs; x MFF, autologous, micro-fragmented fat; x pBSCs, autologous, activated peripheral blood stem cells. y P-MSCs, allogeneic, placental MSCs.

Abbreviations of treatments (in alphabetical order): AD, arthroscopic debridement; BMACBMAC; bone marrow concentrate; C, cells; CM, conservative management; CS, corticosteroid; HA, hyluronic acid; HTO, high tibial osteotomy; MACI, matrix-induced, autologous chondrocyte implant; MF, microfracture; OA, oral acetaminophen; PPP, platelet poor plasma; PRP, platelet rich plasma; RS, Ringer solution; U, unknown.

								Reference no. in the main text																			
R	C	T	First author	Year	Cells	T	Control	15	16	17	18	19	20	21	22	23	24	25	26	27	28	29	30	31	32	33	N
[12]	1	a	Cherian	2015	y Ch-TGFß	C	Saline																●				1
[13]	1	a	Gupta	2016	y BM-MSCs	C + HA	S + HA		●	●	●			●	●		●	●				●	●	●	●		11
[14]	1	a	Emadedin	2018	x BM-MSCs	C	Saline		●	●			●	●	●		●			●	●	●	●		●		11
[15]	1	a	Kim	2018	y Ch-TGFß	C	Saline																●	●			2
[16]	1	a	Kuah	2018	y ADSCs	C	Saline			●	●	●		●	●		●				●	●	●		●		10
[17]	1	a	Khalifeh Soltani	2019	y P-MSCs	C	Saline			●												●			●		3
[18]	1	a	Lee	2019	x ADSCs	C	Saline			●	●	●		●			●		●	●	●		●	●	●		11
[19]	1	a	Garza	2020	x ADRCs	C	RS									●	●		●						●		4
[20]	2	a	Vega	2015	y BM-MSCs	C	HA	●	●	●	●	●		●	●		●	●		●	●	●	●	●	●		15
[21]	2	a	Wang	2016	y hUC-MSCs	C	HA	●			●												●				3
[22]	2	a	Goncars	2017	x BMACBMAC	C	HA														●						1
[23]	2	a	Lu	2019	x ADSCs	C	HA	●		●					●		●	●		●	●		●	●	●	●	11
[24]	2	a	Matas	2019	y hUC-MSCs	C	HA	●		●	●		●	●	●								●		●		8
[25]	2	a	Anz	2020	x BMACBMAC	C	PRP									●											1
[28]	3	a	Garay-Mendoza	2018	x BMACBMAC	C	OA									●	●				●						3
[29]	3	a	Song	2018	x ADSCs	C	C				●								●								2
[30]	3	a	Freitag	2019	x ADSCs	C	CM			●							●	●	●		●		●		●		7
[32]	3	a	Bastos	2020	x BM-MSCs	C	C + PRP +CS						●				●				●						2
[37]	3	c	Jo	2014	x ADSCs	C	C												●								1
[38]	3	c	Pers	2016	x ADSCs	C	C				●								●								2
[39]	3	c	Jo	2017	x ADSCs	C	C				●								●								2
[40]	3	c	Pers	2018	x ADSCs	C	C														●						1
[46]	3	d	Mautner	2019	x MFF	C	BMACBMAC									●											1

Table S3 (cont.)

R	C	T	First author	Year	Cells	T	Control	Reference no. in the main text																			
								15	16	17	18	19	20	21	22	23	24	25	26	27	28	29	30	31	32	33	N
[47]	3	d	Yokota	2019	x ADRCs	C	C									●			●								2
[50]	4	a	Varma	2010	x BM-MSCs	C + AD	AD		●																		1
[51]	4	a	Wong	2013	x BM-MSCs	C + MF + HTO + HA	MF + HTO + HA					●					●	●									3
[52]	4	a	Koh	2014	x ADRCs	C + AD + HTO + PRP	AD + HTO + PRP										●							●			2
[53]	4	a	Lamo-Espinosa	2016	x BM-MSCs	C + HA	HA	●	●	●		●		●	●		●	●		●		●	●		●		12
[55]	4	a	Bastos	2018	x BM-MSCs	C + PRP	C				●												●				3
[56]	4	a	Zhang	2018	x ADSCs	C + HA	C													●							1
[62]	4	b	Shapiro	2017	x BMACBMAC	C + PPP	Saline					●															1
[63]	4	b	Hong	2019	x ADRCs	C + AD	HA + AD										●		●						●	●	4
[64]	4	b	Shapiro	2019	x BMACBMAC	C + PPP	Saline																		●		1
[65]	4	c	Koh	2012	x ADSCs	C + AD + PRP	AD + PRP				●						●	●						●			4
[67]	4	c	Nguyen	2017	x ADRCs	C + AD + MF + PRP	AD + MF												●								1
[69]	4	c	Tran	2019	x ADRCs	C + AD + MF	AD + MF										●		●								2
[81]	5	e	Koh	2013	x ADRCs	C + PRP + HA	None												●								1
[82]	5	e	Orozco	2013	x BM-MSCs	C	None														●						1
[98]	5	e	Soler	2016	x BM-MSCs	C	None														●						1
[99]	5	e	Al-Najar	2017	x BM-MSCs	C	None														●						1
[100]	5	e	Hudetz	2017	x MFF	C	None														●						1
[105]	5	e	Yokota	2017	x ADRCs	C	None									●					●						2
[110]	5	e	Shaw	2018	x BMACBMAC	C	None														●						1
[111]	5	e	Spasovski	2018	x ADSCs	C	None				●								●								2
[112]	5	e	Themistocleous	2018	x BMACBMAC	C	None									●											1
[115]	5	e	Hudetz	2019	x MFF	C	None									●			●		●						3
[118]	5	e	Roato	2019	x CLL	C + AD	None												●								1
[119]	5	e	Schiavone Panni	2019	x MFF	C + AD	None												●								1
[137]	5	e	Tsubosaka	2020	x ADRCs	C	None									●					●						2
[156]	6	a	Saw	2013	x pBSCs	C + MF + HA	HA					●															1
[157]	6	a	Akgun	2015	x S-MSCs	C in Sc + AD	MACI																	●			1
[161]	6	a	Hashimoto	2019	x BM-MSCs	C + MF	MF																	●			1
[218]	6	a	Vangsness	2014	y BM-MSCs				●								●	●									3
[219]	1	a	Bhattacharia	2010	---							●															1
[220]			Lv	2015	U												●										1
[221]			Ha	2018	U				●																		1

Table S4 | Details of the sub-analyses performed in a meta-analysis by Jiang et al. [199].

Abbreviations: R, reference number; C, category of study as outlined in Table 2 in the main text; T, type of study as outlined in Table 3 in the main text; Y, year of publication; O, origin of cells (allo, allogeneic cells; auto; autologous cells); QC, quality criteria (outlined in Table 6 in the main text; a point indicates that the corresponding quality criterion was fulfilled). The abbreviations of the cell types and treatments are provided in Table S1.

WOMAC Total score reported at 6 months post treatment

**R**	**C**	**T**	**First author**	**Y**	**Cells**	**Treatment**	**Control**	**O**
[24]	2	a	Matas	2019	hUC-MSCs	C	HA	allo
[20]	2	a	Vega	2015	BM-MSCs	C	HA	allo
[23]	2	a	Lu	2019	ADSCs	C	HA	auto
[21]	2	a	Wang	2016	hUC-MSCs	C	HA	allo

**QC**	**1**	**2**	**3**	**4**	**5**	**6**	**7**	**8**
	●	●	●	●			●	●

WOMAC Total score reported at 12 months post treatment

**R**	**C**	**T**	**First author**	**Y**	**Cells**	**Treatment**	**Control**	**O**
[24]	2	a	Matas	2019	hUC-MSCs	C	HA	allo
[20]	2	a	Vega	2015	BM-MSCs	C	HA	allo
[23]	2	a	Lu	2019	ADSCs	C	HA	auto

**QC**	**1**	**2**	**3**	**4**	**5**	**6**	**7**	**8**
	●	●	●	●			●	●

WOMAC Pain score reported at 6 months post treatment

**R**	**C**	**T**	**First author**	**Y**	**Cells**	**Treatment**	**Control**	**O**
[53]	4	a	Lamo-Espinosa	2016	BM-MSCs	C + HA	HA	auto
[23]	2	a	Lu	2019	ADSCs	C	HA	auto
[24]	2	a	Matas	2019	hUC-MSCs	C	HA	allo
[20]	2	a	Vega	2015	BM-MSCs	C	HA	allo

**QC**	**1**	**2**	**3**	**4**	**5**	**6**	**7**	**8**
	●	●	●	●			●	●

WOMAC Stiffness score reported at 6 months post treatment

**R**	**C**	**T**	**First author**	**Y**	**Cells**	**Treatment**	**Control**	**O**
[23]	2	a	Lu	2019	ADSCs	C	HA	auto
[24]	2	a	Matas	2019	hUC-MSCs	C	HA	allo

**QC**	**1**	**2**	**3**	**4**	**5**	**6**	**7**	**8**
	●	●	●	●			●	●

WOMAC Stiffness score reported at 12 months post treatment

R	C	T	First author	Y	Cells	Treatment	Control	O
[23]	2	a	Lu	2019	ADSCs	C	HA	auto
[24]	2	a	Matas	2019	hUC-MSCs	C	HA	allo

QC	1	2	3	4	5	6	7	8
	●	●	●	●			●	●

WOMAC Function score reported at 6 months post treatment

R	C	T	First author	Y	Cells	Treatment	Control	O
[23]	2	a	Lu	2019	ADSCs	C	HA	auto
[24]	2	a	Matas	2019	hUC-MSCs	C	HA	allo

QC	1	2	3	4	5	6	7	8
	●	●	●	●			●	●

WOMAC Function score reported at 12 months post treatment

R	C	T	First author	Y	Cells	Treatment	Control	O
[23]	2	a	Lu	2019	ADSCs	C	HA	auto
[24]	2	a	Matas	2019	hUC-MSCs	C	HA	allo

QC	1	2	3	4	5	6	7	8
	●	●	●	●			●	●

VAS Pain score reported at 6 months post treatment

R	C	T	First author	Y	Cells	Treatment	Control	O
[53]	4	a	Lamo-Espinosa	2016	BM-MSCs	C + HA	HA	auto
[23]	2	a	Lu	2019	ADSCs	C	HA	auto
[24]	2	a	Matas	2019	hUC-MSCs	C	HA	allo
[20]	2	a	Vega	2015	BM-MSCs	C	HA	allo

QC	1	2	3	4	5	6	7	8
	●	●	●	●			●	●

VAS pain score reported at 12 months post treatment

R	C	T	First author	Y	Cells	Treatment	Control	O
[53]	4	a	Lamo-Espinosa	2016	BM-MSCs	C + HA	HA	auto
[23]	2	a	Lu	2019	ADSCs	C	HA	auto
[24]	2	a	Matas	2019	hUC-MSCs	C	HA	allo
[20]	2	a	Vega	2015	BM-MSCs	C	HA	allo

QC	1	2	3	4	5	6	7	8
	●	●	●	●			●	●

WORMS score reported at 6 months post treatment

R	C	T	First author	Y	Cells	Treatment	Control	O
[53]	4	a	Lamo-Espinosa	2016	BM-MSCs	C + HA	HA	auto
[24]	2	a	Matas	2019	hUC-MSCs	C	HA	allo

QC	1	2	3	4	5	6	7	8
	●	●	●	●			●	●

WORMS score reported at 12 months post treatment

R	C	T	First author	Y	Cells	Treatment	Control	O
[53]	4	a	Lamo-Espinosa	2016	BM-MSCs	C + HA	HA	auto
[24]	2	a	Matas	2019	hUC-MSCs	C	HA	allo

QC	1	2	3	4	5	6	7	8
	●	●	●	●			●	●

LEQUESNE score reported at 6 months post treatment

R	C	T	First author	Y	Cells	Treatment	Control	O
[53]	4	a	Lamo-Espinosa	2016	BM-MSCs	C + HA	HA	auto
[20]	2	a	Vega	2015	BM-MSCs	C	HA	allo

QC	1	2	3	4	5	6	7	8
	●	●	●	●			●	●

LEQUESNE score reported at 12 months post treatment

R	C	T	First author	Y	Cells	Treatment	Control	O
[53]	4	a	Lamo-Espinosa	2016	BM-MSCs	C + HA	HA	auto
[20]	2	a	Vega	2015	BM-MSCs	C	HA	allo

QC	1	2	3	4	5	6	7	8
	●	●	●	●			●	●

Table S5 | Details of the sub-analyses performed in a meta-analysis by Qu and Sun [200].

Abbreviations: R, reference number; C, category of study as outlined in Table 2 in the main text; T, type of study as outlined in Table 3 in the main text; Y, year of publication; O, origin of cells (allo, allogeneic cells; auto; autologous cells); QC, quality criteria (outlined in Table 6 in the main text; a point indicates that the corresponding quality criterion was fulfilled). The abbreviations of the cell types and treatments are provided in Table S1.

VAS Pain score reported at 3 months post treatment

R	C	T	First author	Y	Cells	Treatment	Control	O
[15]	4	a	Gupta	2016	BM-MSCs	C + HA	S + HA	allo
[15]	4	a	Gupta	2016	BM-MSCs	C + HA	S + HA	allo
[15]	4	a	Gupta	2016	BM-MSCs	C + HA	S + HA	allo
[15]	4	a	Gupta	2016	BM-MSCs	C + HA	S + HA	allo
[50]	4	a	Varma	2010	BM-MSCs	C + AD	AD	?
[221]			Ha	2018				

QC	1	2	3	4	5	6	7	8
	●	●	●	●	●	●	●	

VAS Pain score reported at 6 months post treatment

R	C	T	First author	Y	Cells	Treatment	Control	O
[14]	1	a	Emadedin	2018	BM-MSCs	C	S	auto
[15]	1	a	Gupta	2016	BM-MSCs	C	S	allo
[15]	4	a	Gupta	2016	BM-MSCs	C + HA	S + HA	allo
[15]	4	a	Gupta	2016	BM-MSCs	C + HA	S + HA	allo
[15]	4	a	Gupta	2016	BM-MSCs	C + HA	S + HA	allo
[50]	4	a	Varma	2010	BM-MSCs	C + AD	AD	?
[218]	6	a	Vangsness	2014	BM-MSCs	C	S	allo
[218]	6	a	Vangsness	2014	BM-MSCs	C	S	allo
[221]			Ha	2018				

QC	1	2	3	4	5	6	7	8
	●		●	●	●		●	

VAS Pain score reported at 12 months post treatment

R	C	T	First author	Y	Cells	Treatment	Control	O
[15]	4	a	Gupta	2016	BM-MSCs	C + HA	S + HA	allo
[15]	4	a	Gupta	2016	BM-MSCs	C + HA	S + HA	allo
[15]	4	a	Gupta	2016	BM-MSCs	C + HA	S + HA	allo
[15]	4	a	Gupta	2016	BM-MSCs	C + HA	S + HA	allo
[50]	4	a	Varma	2010	BM-MSCs	C + AD	AD	?
[20]	2	a	Vega	2015	BM-MSCs	C	HA	allo
[218]	6	a	Vangsness	2014	BM-MSCs	C	S	allo
[218]	6	a	Vangsness	2014	BM-MSCs	C	S	allo
[221]			Ha	2018				

QC	1	2	3	4	5	6	7	8
	●		●	●		●	●	

VAS Pain score reported at 24 months post treatment

R	C	T	First author	Y	Cells	Treatment	Control	O
[218]	6	a	Vangsness	2014	BM-MSCs	C	S	allo
[218]	6	a	Vangsness	2014	BM-MSCs	C	S	allo

QC	1	2	3	4	5	6	7	8
	●		●	●	●	●	●	

WOMAC Pain score reported at 3 months post treatment

R	C	T	First author	Y	Cells	Treatment	Control	O
[15]	4	a	Gupta	2016	BM-MSCs	C	S + HA	allo
[15]	4	a	Gupta	2016	BM-MSCs	C	S + HA	allo
[15]	4	a	Gupta	2016	BM-MSCs	C	S + HA	allo
[15]	4	a	Gupta	2016	BM-MSCs	C	S + HA	allo

QC	1	2	3	4	5	6	7	8
	●	●	●	●	●	●	●	

WOMAC Pain score reported at 6 months post treatment

R	C	T	First author	Y	Cells	Treatment	Control	O
[14]	1	a	Emadedin	2018	BM-MSCs	C	S	auto
[53]	4	a	Lamo-Espinosa	2016	BM-MSCs	C + HA	HA	auto
[53]	4	a	Lamo-Espinosa	2016	BM-MSCs	C + HA	HA	auto
[15]	4	a	Gupta	2016	BM-MSCs	C + HA	S + HA	allo
[15]	4	a	Gupta	2016	BM-MSCs	C + HA	S + HA	allo
[15]	4	a	Gupta	2016	BM-MSCs	C + HA	S + HA	allo
[15]	4	a	Gupta	2016	BM-MSCs	C + HA	S + HA	allo

QC	1	2	3	4	5	6	7	8
	●	●	●	●	●		●	

WOMAC Pain score reported at 12 months post treatment

R	C	T	First author	Y	Cells	Treatment	Control	O
[53]	4	a	Lamo-Espinosa	2016	BM-MSCs	C + HA	HA	auto
[53]	4	a	Lamo-Espinosa	2016	BM-MSCs	C + HA	HA	auto
[15]	4	a	Gupta	2016	BM-MSCs	C + HA	S + HA	allo
[15]	4	a	Gupta	2016	BM-MSCs	C + HA	S + HA	allo
[15]	4	a	Gupta	2016	BM-MSCs	C + HA	S + HA	allo
[15]	4	a	Gupta	2016	BM-MSCs	C + HA	S + HA	allo
[20]	2	a	Vega	2015	BM-MSCs	C + HA	HA	allo

QC	1	2	3	4	5	6	7	8
	●	●	●	●			●	

Table S6 | Details of the sub-analyses performed in a meta-analysis by Ma et al. [201].

Abbreviations: R, reference number; C, category of study as outlined in Table 2 in the main text; T, type of study as outlined in Table 3 in the main text; Y, year of publication; O, origin of cells (allo, allogeneic cells; auto; autologous cells); QC, quality criteria (outlined in Table 6 in the main text; a point indicates that the corresponding quality criterion was fulfilled). The abbreviations of the cell types and treatments are provided in Table S1.

VAS Pain score

R	C	T	First author	Y	Cells	Treatment	Control	O
[14]	1	a	Emadedin	2018	BM-MSCs	C	S	auto
[15]	4	a	Gupta	2016	BM-MSCs	C + HA	S + HA	allo
[16]	1	a	Kuah	2018	ADSCs	C	S	allo
[53]	4	a	Lamo-Espinosa	2016	BM-MSCs	C + HA	HA	auto
[23]	2	a	Lu	2019	ADSCs	C	HA	auto
[24]	2	a	Matas	2019	hUC-MSCs	C	HA	allo
[20]	2	a	Vega	2015	BM-MSCs	C	HA	allo

QC	1	2	3	4	5	6	7	8
	●	●	●	●			●	●

WOMAC Total score

R	C	T	First author	Y	Cells	Treatment	Control	O
[14]	1	a	Emadedin	2018	BM-MSCs	C	S	auto
[30]	3	a	Freitag	2019	ADSCs	C	CM	auto
[15]	4	a	Gupta	2016	BM-MSCs	C + HA	S + HA	allo
[53]	4	a	Lamo-Espinosa	2016	BM-MSCs	C + HA	HA	auto
[23]	2	a	Lu	2019	ADSCs	C	HA	auto
[24]	2	a	Matas	2019	hUC-MSCs	C	HA	allo
[20]	2	a	Vega	2015	BM-MSCs	C	HA	allo

QC	1	2	3	4	5	6	7	8
	●	●	●	●			●	●

WOMAC Pain score

R	C	T	First author	Y	Cells	Treatment	Control	O
[14]	1	a	Emadedin	2018	BM-MSCs	C	S	auto
[15]	4	a	Gupta	2016	BM-MSCs	C + HA	S + HA	allo
[16]	1	a	Kuah	2018	ADSCs	C	S	allo
[53]	4	a	Lamo-Espinosa	2016	BM-MSCs	C + HA	HA	auto
[23]	2	a	Lu	2019	ADSCs	C	HA	auto
[24]	2	a	Matas	2019	hUC-MSCs	C	HA	allo
[20]	2	a	Vega	2015	BM-MSCs	C	HA	allo

QC	1	2	3	4	5	6	7	8
	●	●	●	●			●	●

WOMAC Stiffness score

R	C	T	First author	Y	Cells	Treatment	Control	O
[14]	1	a	Emadedin	2018	BM-MSCs	C	S	auto
[15]	4	a	Gupta	2016	BM-MSCs	C + HA	S + HA	allo
[53]	4	a	Lamo-Espinosa	2016	BM-MSCs	C + HA	HA	auto
[23]	2	a	Lu	2019	ADSCs	C	HA	auto
[24]	2	a	Matas	2019	hUC-MSCs	C	HA	allo

QC	1	2	3	4	5	6	7	8
	●	●	●	●			●	●

WOMAC Function score

R	C	T	First author	Y	Cells	Treatment	Control	O
[14]	1	a	Emadedin	2018	BM-MSCs	C	S	auto
[15]	4	a	Gupta	2016	BM-MSCs	C + HA	S + HA	allo
[53]	4	a	Lamo-Espinosa	2016	BM-MSCs	C + HA	HA	auto
[23]	2	a	Lu	2019	ADSCs	C	HA	auto
[24]	2	a	Matas	2019	hUC-MSCs	C	HA	allo

QC	1	2	3	4	5	6	7	8
	●	●	●	●			●	●

WORMS score

R	C	T	First author	Y	Cells	Treatment	Control	O
[15]	4	a	Gupta	2016	BM-MSCs	C + HA	S + HA	allo
[53]	4	a	Lamo-Espinosa	2016	BM-MSCs	C + HA	HA	auto
[24]	2	a	Matas	2019	hUC-MSCs	C	HA	allo

QC	1	2	3	4	5	6	7	8
	●	●	●	●			●	●

Cartilage volume

R	C	T	First author	Y	Cells	Treatment	Control	O
[16]	1	a	Kuah	2018	ADSCs	C	S	allo
[18]	1	a	Lee	2019	ADSCs	C	S	auto
[23]	2	a	Lu	2019	ADSCs	C	HA	auto

QC	1	2	3	4	5	6	7	8
	●	●	●	●			●	●

Number of patients with adverse events

R	C	T	First author	Y	Cells	Treatment	Control	O
[15]	4	a	Gupta	2016	BM-MSCs	C + HA	S + HA	allo
[17]	1	a	Khalifeh Soltani	2019	P-MSCs	C	S	allo
[16]	1	a	Kuah	2018	ADSCs	C	S	allo
[53]	4	a	Lamo-Espinosa	2016	BM-MSCs	C + HA	HA	auto
[18]	1	a	Lee	2019	ADSCs	C	S	auto
[23]	2	a	Lu	2019	ADSCs	C	HA	auto

QC	1	2	3	4	5	6	7	8
	●	●	●	●			●	●

Table S7 | Details of the sub-analyses performed in a meta-analysis by Huang et al. [203].

Abbreviations: R, reference number; C, category of study as outlined in Table 2 in the main text; T, type of study as outlined in Table 3 in the main text; Y, year of publication; O, origin of cells (allo, allogeneic cells; auto; autologous cells); QC, quality criteria (outlined in Table 6 in the main text; a point indicates that the corresponding quality criterion was fulfilled). The abbreviations of the cell types and treatments are provided in Table S1.

VAS Pain score reported at 3 months post treatment

R	C	T	First author	Y	Cells	Treatment	Control	O
[219]			Bhattacharia	2010				
[16]	1	a	Kuah	2018	ADSCs	C	S	allo
[16]	1	a	Kuah	2018	ADSCs	C	S	allo
[53]	4	a	Lamo-Espinosa	2016	BM-MSCs	C + HA	HA	auto
[53]	4	a	Lamo-Espinosa	2016	BM-MSCs	C + HA	HA	auto
[62]	4	b	Shapiro	2017	BMACBMAC	C + PPP	S	auto

QC	1	2	3	4	5	6	7	8
	●	●						

VAS Pain score reported at 6 months post treatment

R	C	T	First author	Y	Cells	Treatment	Control	O
[219]			Bhattacharia	2010				
[16]	1	a	Kuah	2018	ADSCs	C	S	allo
[16]	1	a	Kuah	2018	ADSCs	C	S	allo
[53]	4	a	Lamo-Espinosa	2016	BM-MSCs	C + HA	HA	auto
[53]	4	a	Lamo-Espinosa	2016	BM-MSCs	C + HA	HA	auto
[62]	4	b	Shapiro	2017	BMACBMAC	C + PPP	S	auto

QC	1	2	3	4	5	6	7	8
	●	●						

VAS pain score reported at 12 months post treatment

R	C	T	First author	Y	Cells	Treatment	Control	O
[16]	1	a	Kuah	2018	ADSCs	C	S	allo
[16]	1	a	Kuah	2018	ADSCs	C	S	allo
[53]	4	a	Lamo-Espinosa	2016	BM-MSCs	C + HA	HA	auto
[53]	4	a	Lamo-Espinosa	2016	BM-MSCs	C + HA	HA	auto
[62]	4	b	Shapiro	2017	BMACBMAC	C + PPP	S	auto
[20]	2	a	Vega	2015	BM-MSCs	C	HA	allo

QC	1	2	3	4	5	6	7	8
	●	●	●					

WOMAC Pain score reported at 3 months post treatment

R	C	T	First author	Y	Cells	Treatment	Control	O
[16]	1	a	Kuah	2018	ADSCs	C	S	allo
[16]	1	a	Kuah	2018	ADSCs	C	S	allo
[53]	4	a	Lamo-Espinosa	2016	BM-MSCs	C + HA	HA	auto
[53]	4	a	Lamo-Espinosa	2016	BM-MSCs	C + HA	HA	auto

QC	1	2	3	4	5	6	7	8
	●	●	●	●	●		●	

WOMAC Pain score reported at 6 months post treatment

R	C	T	First author	Y	Cells	Treatment	Control	O
[16]	1	a	Kuah	2018	ADSCs	C	S	allo
[16]	1	a	Kuah	2018	ADSCs	C	S	allo
[53]	4	a	Lamo-Espinosa	2016	BM-MSCs	C + HA	HA	auto
[53]	4	a	Lamo-Espinosa	2016	BM-MSCs	C + HA	HA	auto

QC	1	2	3	4	5	6	7	8
	●	●	●	●	●		●	

WOMAC Pain score reported at 12 months post treatment

R	C	T	First author	Y	Cells	Treatment	Control	O
[16]	1	a	Kuah	2018	ADSCs	C	S	allo
[53]	4	a	Lamo-Espinosa	2016	BM-MSCs	C + HA	HA	auto
[53]	4	a	Lamo-Espinosa	2016	BM-MSCs	C + HA	HA	auto
[20]	2	a	Vega	2015	BM-MSCs	C	HA	allo

QC	1	2	3	4	5	6	7	8
	●	●	●	●	●		●	

WOMAC Stiffness score reported at 3 months post treatment

R	C	T	First author	Y	Cells	Treatment	Control	O
[16]	1	a	Kuah	2018	ADSCs	C	S	allo
[16]	1	a	Kuah	2018	ADSCs	C	S	allo
[53]	4	a	Lamo-Espinosa	2016	BM-MSCs	C + HA	HA	auto
[53]	4	a	Lamo-Espinosa	2016	BM-MSCs	C + HA	HA	auto

QC	1	2	3	4	5	6	7	8
	●	●	●	●	●		●	

WOMAC Stiffnes score reported at 6 months post treatment

R	C	T	First author	Y	Cells	Treatment	Control	O
[16]	1	a	Kuah	2018	ADSCs	C	S	allo
[16]	1	a	Kuah	2018	ADSCs	C	S	allo
[53]	4	a	Lamo-Espinosa	2016	BM-MSCs	C + HA	HA	auto
[53]	4	a	Lamo-Espinosa	2016	BM-MSCs	C + HA	HA	auto

QC	1	2	3	4	5	6	7	8
	●	●	●	●	●		●	

WOMAC Stiffness score reported at 12 months post treatment

R	C	T	First author	Y	Cells	Treatment	Control	O
[16]	1	a	Kuah	2018	ADSCs	C	S	allo
[16]	1	a	Kuah	2018	ADSCs	C	S	allo
[53]	4	a	Lamo-Espinosa	2016	BM-MSCs	C + HA	HA	auto
[53]	4	a	Lamo-Espinosa	2016	BM-MSCs	C + HA	HA	auto

QC	1	2	3	4	5	6	7	8
	●	●	●	●	●		●	

WOMAC Function score reported at 3 months post treatment

R	C	T	First author	Y	Cells	Treatment	Control	O
[16]	1	a	Kuah	2018	ADSCs	C	S	allo
[16]	1	a	Kuah	2018	ADSCs	C	S	allo
[53]	4	a	Lamo-Espinosa	2016	BM-MSCs	C + HA	HA	auto
[53]	4	a	Lamo-Espinosa	2016	BM-MSCs	C + HA	HA	auto

QC	1	2	3	4	5	6	7	8
	●	●	●	●	●		●	

WOMAC Function score reported at 6 months post treatment

R	C	T	First author	Y	Cells	Treatment	Control	O
[16]	1	a	Kuah	2018	ADSCs	C	S	allo
[16]	1	a	Kuah	2018	ADSCs	C	S	allo
[53]	4	a	Lamo-Espinosa	2016	BM-MSCs	C + HA	HA	auto
[53]	4	a	Lamo-Espinosa	2016	BM-MSCs	C + HA	HA	auto

QC	1	2	3	4	5	6	7	8
	●	●	●	●	●		●	

WOMAC Function score reported at 12 months post treatment

R	C	T	First author	Y	Cells	Treatment	Control	O
[16]	1	a	Kuah	2018	ADSCs	C	S	allo
[16]	1	a	Kuah	2018	ADSCs	C	S	allo
[53]	4	a	Lamo-Espinosa	2016	BM-MSCs	C + HA	HA	auto
[53]	4	a	Lamo-Espinosa	2016	BM-MSCs	C + HA	HA	auto

QC	1	2	3	4	5	6	7	8
	●	●	●	●	●		●	

International Knee Documentation Committee (IKDC) score reported at 6 months post treatment

R	C	T	First author	Y	Cells	Treatment	Control	O
[156]	6	a	Saw	2013	pBSCs	C + MF + HA	MF + HA	auto
[51]	4	a	Wong	2013	BM-MSCs	C + MF + HTO + HA	MF + HTO + HA	auto

QC	1	2	3	4	5	6	7	8
	●		●	●	●	●	●	●

International Knee Documentation Committee (IKDC) score reported at 12 months post treatment

R	C	T	First author	Y	Cells	Treatment	Control	O
[156]	6	a	Saw	2013	pBSCs	C + MF + HA	MF + HA	auto
[51]	4	a	Wong	2013	BM-MSCs	C + MF + HTO + HA	MF + HTO + HA	auto

QC	1	2	3	4	5	6	7	8
	●		●	●	●	●	●	●

International Knee Documentation Committee (IKDC) score reported at 24 months post treatment

R	C	T	First author	Y	Cells	Treatment	Control	O
[156]	6	a	Saw	2013	pBSCs	C + MF + HA	MF + HA	auto
[51]	4	a	Wong	2013	BM-MSCs	C + MF + HTO + HA	MF + HTO + HA	auto

QC	1	2	3	4	5	6	7	8
	●		●	●	●	●	●	●

Adverse events

R	C	T	First author	Y	Cells	Treatment	Control	O
[16]	1	a	Kuah	2018	ADSCs	C	S	allo
[16]	1	a	Kuah	2018	ADSCs	C	S	allo
[18]	1	a	Lee	2019	ADSCs	C	S	auto

QC	1	2	3	4	5	6	7	8
	●	●	●	●	●		●	●

Table S8 | Details of the sub-analyses performed in a meta-analysis by Wei et al. [205].

Abbreviations: R, reference number; C, category of study as outlined in Table 2 in the main text; T, type of study as outlined in Table 3 in the main text; Y, year of publication; O, origin of cells (allo, allogeneic cells; auto; autologous cells); QC, quality criteria (outlined in Table 6 in the main text; a point indicates that the corresponding quality criterion was fulfilled). The abbreviations of the cell types and treatments are provided in Table S1.

VAS Pain score

R	C	T	First author	Y	Cells	Treatment	Control	O
[20]	2	a	Vega	2015	BM-MSCs	C	HA	allo
[15]	4	a	Gupta	2016	BM-MSCs	C + HA	S + HA	allo
[53]	4	a	Lamo-Espinosa	2016	BM-MSCs	C + HA	HA	auto
[14]	1	a	Emadedin	2018	BM-MSCs	C	S	auto
[16]	1	a	Kuah	2018	ADSCs	C	S	allo
[24]	2	a	Matas	2019	hUC-MSCs	C	HA	allo
[18]	1	a	Lee	2019	ADSCs	C	S	auto

QC	1	2	3	4	5	6	7	8
	●	●	●	●			●	●

Functional improvement

R	C	T	First author	Y	Cells	Treatment	Control	O
[20]	2	a	Vega	2015	BM-MSCs	C	HA	allo
[15]	4	a	Gupta	2016	BM-MSCs	C + HA	S + HA	allo
[53]	4	a	Lamo-Espinosa	2016	BM-MSCs	C + HA	HA	auto
[14]	1	a	Emadedin	2018	BM-MSCs	C	S	auto
[16]	1	a	Kuah	2018	ADSCs	C	S	allo
[24]	2	a	Matas	2019	hUC-MSCs	C	HA	allo
[18]	1	a	Lee	2019	ADSCs	C	S	auto

QC	1	2	3	4	5	6	7	8
	●	●	●	●			●	●

Structural assessment

R	C	T	First author	Y	Cells	Treatment	Control	O
[15]	4	a	Gupta	2016	BM-MSCs	C + HA	S + HA	allo
[53]	4	a	Lamo-Espinosa	2016	BM-MSCs	C + HA	HA	auto
[16]	1	a	Kuah	2018	ADSCs	C	S	allo
[24]	2	a	Matas	2019	hUC-MSCs	C	HA	allo
[18]	1	a	Lee	2019	ADSCs	C	S	auto

QC	1	2	3	4	5	6	7	8
	●	●	●	●			●	●

Table S9 | Details of the sub-analyses performed in a meta-analysis by Wang et al. [206].

Abbreviations: R, reference number; C, category of study as outlined in Table 2 in the main text; T, type of study as outlined in Table 3 in the main text; Y, year of publication; O, origin of cells (allo, allogeneic cells; auto; autologous cells); QC, quality criteria (outlined in Table 6 in the main text; a point indicates that the corresponding quality criterion was fulfilled). The abbreviations of the cell types and treatments are provided in Table S1.

Adverse events

R	C	T	First author	Y	Cells	Treatment	Control	O
[15]	4	a	Gupta	2016	BM-MSCs	C + HA	S + HA	allo
[16]	1	a	Kuah	2018	ADSCs	C	S	allo
[23]	2	a	Lu	2019	ADSCs	C	HA	auto
[24]	2	a	Matas	2019	hUC-MSCs	C	HA	allo
[20]	2	a	Vega	2015	BM-MSCs	C	HA	allo

QC	1	2	3	4	5	6	7	8
	●	●	●	●			●	●

VAS score low dose

R	C	T	First author	Y	Cells	Treatment	Control	O
[15]	4	a	Gupta	2016	BM-MSCs	C + HA	S + HA	allo
[16]	1	a	Kuah	2018	ADSCs	C	S	allo
[53]	4	a	Lamo-Espinosa	2016	BM-MSCs	C + HA	HA	auto
[24]	2	a	Matas	2019	hUC-MSCs	C	HA	allo

QC	1	2	3	4	5	6	7	8
	●	●	●	●			●	●

VAS score medium dose

R	C	T	First author	Y	Cells	Treatment	Control	O
[14]	1	a	Emadedin	2018	BM-MSCs	C	S	auto
[20]	2	a	Vega	2015	BM-MSCs	C	HA	allo

QC	1	2	3	4	5	6	7	8
	●	●	●	●			●	●

VAS scire high dose

R	C	T	First author	Y	Cells	Treatment	Control	O
[15]	4	a	Gupta	2016	BM-MSCs	C + HA	S + HA	allo
[53]	4	a	Lamo-Espinosa	2016	BM-MSCs	C + HA	HA	auto
[23]	2	a	Lu	2019	ADSCs	C	HA	auto

QC	1	2	3	4	5	6	7	8
	●	●	●	●			●	●

WOMAC Pain score low dose

R	C	T	First author	Y	Cells	Treatment	Control	O
[15]	4	a	Gupta	2016	BM-MSCs	C + HA	S + HA	allo
[16]	1	a	Kuah	2018	ADSCs	C	S	allo
[53]	4	a	Lamo-Espinosa	2016	BM-MSCs	C + HA	HA	auto
[24]	2	a	Matas	2019	hUC-MSCs	C	HA	allo

QC	1	2	3	4	5	6	7	8
	●	●	●	●			●	●

WOMAC Pain score medium dose

R	C	T	First author	Y	Cells	Treatment	Control	O
[14]	1	a	Emadedin	2018	BM-MSCs	C	S	auto
[20]	2	a	Vega	2015	BM-MSCs	C	HA	allo

QC	1	2	3	4	5	6	7	8
	●	●	●	●			●	●

WOMAC Pain score high dose

R	C	T	First author	Y	Cells	Treatment	Control	O
[15]	4	a	Gupta	2016	BM-MSCs	C + HA	S + HA	allo
[53]	4	a	Lamo-Espinosa	2016	BM-MSCs	C + HA	HA	auto
[23]	2	a	Lu	2019	ADSCs	C	HA	auto

QC	1	2	3	4	5	6	7	8
	●	●	●	●			●	●

WOMAC Stiffness score low dose

R	C	T	First author	Y	Cells	Treatment	Control	O
[15]	4	a	Gupta	2016	BM-MSCs	C + HA	S + HA	allo
[53]	4	a	Lamo-Espinosa	2016	BM-MSCs	C + HA	HA	auto
[24]	2	a	Matas	2019	hUC-MSCs	C	HA	allo

QC	1	2	3	4	5	6	7	8
	●	●	●	●			●	●

WOMAC Stiffness score medium dose

R	C	T	First author	Y	Cells	Treatment	Control	O
[14]	1	a	Emadedin	2018	BM-MSCs	C	S	auto

QC	1	2	3	4	5	6	7	8
		●	●	●	●	●	●	●

WOMAC Stiffness score high dose

R	C	T	First author	Y	Cells	Treatment	Control	O
[15]	4	a	Gupta	2016	BM-MSCs	C + HA	S + HA	allo
[53]	4	a	Lamo-Espinosa	2016	BM-MSCs	C + HA	HA	auto
[23]	2	a	Lu	2019	ADSCs	C	HA	auto

QC	1	2	3	4	5	6	7	8
	●	●	●	●			●	●

WOMAC Physical Function score low dose

R	C	T	First author	Y	Cells	Treatment	Control	O
[15]	4	a	Gupta	2016	BM-MSCs	C + HA	S + HA	allo
[53]	4	a	Lamo-Espinosa	2016	BM-MSCs	C + HA	HA	auto
[24]	2	a	Matas	2019	hUC-MSCs	C	HA	allo

QC	1	2	3	4	5	6	7	8
	●	●	●	●			●	●

WOMAC Physical Function score medium low dose

R	C	T	First author	Y	Cells	Treatment	Control	O
[14]	1	a	Emadedin	2018	BM-MSCs	C	S	auto

QC	1	2	3	4	5	6	7	8
		●	●	●	●	●	●	●

WOMAC Physical Function score high dose

R	C	T	First author	Y	Cells	Treatment	Control	O
[15]	4	a	Gupta	2016	BM-MSCs	C + HA	S + HA	allo
[53]	4	a	Lamo-Espinosa	2016	BM-MSCs	C + HA	HA	auto
[23]	2	a	Lu	2019	ADSCs	C	HA	auto

QC	1	2	3	4	5	6	7	8
	●	●	●	●			●	●

WOMAC Total score low dose

R	C	T	First author	Y	Cells	Treatment	Control	O
[15]	4	a	Gupta	2016	BM-MSCs	C + HA	S + HA	allo
[53]	4	a	Lamo-Espinosa	2016	BM-MSCs	C + HA	HA	auto
[23]	2	a	Matas	2019	hUC-MSCs	C	HA	allo

QC	1	2	3	4	5	6	7	8
	●	●	●	●			●	●

WOMAC Total score medium dose

R	C	T	First author	Y	Cells	Treatment	Control	O
[14]	1	a	Emadedin	2018	BM-MSCs	C	S	auto
[20]	2	a	Vega	2015	BM-MSCs	C	HA	allo

QC	1	2	3	4	5	6	7	8
	●	●	●	●			●	●

WOMAC Total score high dose

R	C	T	First author	Y	Cells	Treatment	Control	O
[15]	4	a	Gupta	2016	BM-MSCs	C + HA	S + HA	allo
[53]	4	a	Lamo-Espinosa	2016	BM-MSCs	C + HA	HA	auto
[23]	2	a	Lu	2019	ADSCs	C	HA	auto

QC	1	2	3	4	5	6	7	8
	●	●	●	●			●	●

Whole-organ MRI (WORMS) score reported at 6 months post treatment

R	C	T	First author	Y	Cells	Treatment	Control	O
[15]	4	a	Gupta	2016	BM-MSCs	C + HA	S+ HA	allo
[53]	4	a	Lamo-Espinosa	2016	BM-MSCs	C + HA	HA	auto
[24]	2	a	Matas	2019	hUC-MSCs	C	HA	allo

QC	1	2	3	4	5	6	7	8
	●	●	●	●			●	●

Whole-organ MRI (WORMS) score reported at 12 months post treatment

R	C	T	First author	Y	Cells	Treatment	Control	O
[15]	4	a	Gupta	2016	BM-MSCs	C + HA	S + HA	allo
[53]	4	a	Lamo-Espinosa	2016	BM-MSCs	C + HA	HA	auto
[24]	2	a	Matas	2019	hUC-MSCs	C	HA	allo

QC	1	2	3	4	5	6	7	8
	●	●	●	●			●	●

VAS Pain score bmsc

R	C	T	First author	Y	Cells	Treatment	Control	O
[14]	1	a	Emadedin	2018	BM-MSCs	C	S	auto
[15]	1	a	Gupta	2016	BM-MSCs	C	S	allo
[53]	4	a	Lamo-Espinosa	2016	BM-MSCs	C + HA	HA	auto
[20]	2	a	Vega	2015	BM-MSCs	C	HA	allo

QC	1	2	3	4	5	6	7	8
	●	●	●	●			●	●

VAS Pain score admsc and ucmsc

R	C	T	First author	Y	Cells	Treatment	Control	O
[16]	1	a	Kuah	2018	ADSCs	C	S	allo
[23]	2	a	Lu	2019	ADSCs	C	HA	auto
[24]	2	a	Matas	2019	hUC-MSCs	C	HA	allo

QC	1	2	3	4	5	6	7	8
	●	●	●	●			●	●

WOMAC Pain score bmsc

R	C	T	First author	Y	Cells	Treatment	Control	O
[14]	1	a	Emadedin	2018	BM-MSCs	C	S	auto
[15]	1	a	Gupta	2016	BM-MSCs	C	S	allo
[53]	4	a	Lamo-Espinosa	2016	BM-MSCs	C + HA	HA	auto
[20]	2	a	Vega	2015	BM-MSCs	C	HA	allo

QC	1	2	3	4	5	6	7	8
	●	●	●	●			●	●

WOMAC Pain score admsc and ucmsc

R	C	T	First author	Y	Cells	Treatment	Control	O
[16]	1	a	Kuah	2018	ADSCs	C	S	allo
[23]	2	a	Lu	2019	ADSCs	C	HA	auto
[24]	2	a	Matas	2019	hUC-MSCs	C	HA	allo

QC	1	2	3	4	5	6	7	8
	●	●	●	●			●	●

VAS Pain score allogenic

R	C	T	First author	Y	Cells	Treatment	Control	O
[15]	1	a	Gupta	2016	BM-MSCs	C	S	allo
[16]	1	a	Kuah	2018	ADSCs	C	S	allo
[24]	2	a	Matas	2019	hUC-MSCs	C	HA	allo
[20]	2	a	Vega	2015	BM-MSCs	C	HA	allo

QC	1	2	3	4	5	6	7	8
	●	●	●	●		●	●	●

VAS Pain score autologous

R	C	T	First author	Y	Cells	Treatment	Control	O
[14]	1	a	Emadedin	2018	BM-MSCs	C	S	auto
[53]	4	a	Lamo-Espinosa	2016	BM-MSCs	C + HA	HA	auto
[23]	2	a	Lu	2019	ADSCs	C	HA	auto

QC	1	2	3	4	5	6	7	8
	●	●	●	●		●	●	●

WOMAC pain score allogenic

R	C	T	First author	Y	Cells	Treatment	Control	O
[15]	1	a	Gupta	2016	BM-MSCs	C	S	allo
[16]	1	a	Kuah	2018	ADSCs	C	S	allo
[24]	2	a	Matas	2019	hUC-MSCs	C	HA	allo
[20]	2	a	Vega	2015	BM-MSCs	C	HA	allo

QC	1	2	3	4	5	6	7	8
	●	●	●	●		●	●	●

WOMAC score autologous

R	C	T	First author	Y	Cells	Treatment	Control	O
[14]	1	a	Emadedin	2018	BM-MSCs	C	S	auto
[53]	4	a	Lamo-Espinosa	2016	BM-MSCs	C + HA	HA	auto
[23]	2	a	Lu	2019	ADSCs	C	HA	auto

QC	1	2	3	4	5	6	7	8
	●	●	●	●		●	●	●

Table S10 | Details of the sub-analyses performed in a meta-analysis by Jeyaraman et al. [208].

Abbreviations: R, reference number; C, category of study as outlined in Table 2 in the main text; T, type of study as outlined in Table 3 in the main text; Y, year of publication; O, origin of cells (allo, allogeneic cells; auto; autologous cells); QC, quality criteria (outlined in Table 6 in the main text; a point indicates that the corresponding quality criterion was fulfilled). The abbreviations of the cell types and treatments are provided in Table S1.

VAS Pain score reported at 6 months post treatment using bone marrow-derived cells

R	C	T	First author	Y	Cells	Treatment	Control	O
[218]	6	a	Vangsness	2014	BM-MSCs	C	S	allo
[218]	6	a	Vangsness	2014	BM-MSCs	C	S	allo
[28]	3	a	Garay-Mendoza	2018	BMACBMAC	C	OA	auto
[53]	4	a	Lamo-Espinosa	2016	BM-MSCs	C + HA	HA	auto
[53]	4	a	Lamo-Espinosa	2016	BM-MSCs	C + HA	HA	auto
[14]	1	a	Emadedin	2018	BM-MSCs	C	S	auto
[15]	4	a	Gupta	2016	BM-MSCs	C + HA	S + HA	allo
[15]	4	a	Gupta	2016	BM-MSCs	C + HA	S + HA	allo
[15]	4	a	Gupta	2016	BM-MSCs	C + HA	S + HA	allo
[15]	4	a	Gupta	2016	BM-MSCs	C + HA	S + HA	allo

QC	1	2	3	4	5	6	7	8
	●		●	●	●			

VAS Pain score reported at 12 months post treatment using bone marrow-derived cells

R	C	T	First author	Y	Cells	Treatment	Control	O
[218]	6	a	Vangsness	2014	BM-MSCs	C	S	allo
[218]	6	a	Vangsness	2014	BM-MSCs	C	S	allo
[53]	4	a	Lamo-Espinosa	2016	BM-MSCs	C + HA	HA	auto
[53]	4	a	Lamo-Espinosa	2016	BM-MSCs	C + HA	HA	auto
[15]	4	a	Gupta	2016	BM-MSCs	C + HA	S + HA	allo
[15]	4	a	Gupta	2016	BM-MSCs	C + HA	S + HA	allo
[15]	4	a	Gupta	2016	BM-MSCs	C + HA	S + HA	allo
[15]	4	a	Gupta	2016	BM-MSCs	C + HA	S + HA	allo

QC	1	2	3	4	5	6	7	8
	●		●	●	●		●	

VAS Pain score reported at 24 months post treatment using bone marrow-derived cells

R	C	T	First author	Y	Cells	Treatment	Control	O
[218]	6	a	Vangsness	2014	BM-MSCs	C	S	allo
[218]	6	a	Vangsness	2014	BM-MSCs	C	S	allo

QC	1	2	3	4	5	6	7	8
	●		●	●	●	●	●	

VAS Pain score reported at 6 months post treatment using adipose-derived cells

R	C	T	First author	Y	Cells	Treatment	Control	O
[16]	1	a	Kuah	2018	ADSCs	C	S	allo
[16]	1	a	Kuah	2018	ADSCs	C	S	allo
[23]	2	a	Lu	2019	ADSCs	C	HA	auto
[18]	1	a	Lee	2019	ADSCs	C	S	auto
[63]	4	b	Hong	2019	ADRCs	C + AD	HA + AD	auto

QC	1	2	3	4	5	6	7	8
	●	●	●				●	

VAS Pain score reported at 12 months post treatment using adipose-derived cells

R	C	T	First author	Y	Cells	Treatment	Control	O
[16]	1	a	Kuah	2018	ADSCs	C	S	allo
[16]	1	a	Kuah	2018	ADSCs	C	S	allo
[23]	2	a	Lu	2019	ADSCs	C	HA	auto
[18]	1	a	Lee	2019	ADSCs	C	S	auto
[30]	3	a	Freitag	2019	ADSCs	C	CM	auto
[30]	3	a	Freitag	2019	ADSCs	C	CM	auto

QC	1	2	3	4	5	6	7	8
	●	●	●	●			●	

VAS Pain score reported at 24 months post treatment using adipose-derived cells

R	C	T	First author	Y	Cells	Treatment	Control	O
[65]	4	c	Koh	2012	ADRCs	C + AD + PRP	AD + PRP	auto
[52]	4	a	Koh	2014	ADRCs	C + AD + HTO + PRP	AD + HTO + PRP	auto
[69]	4	c	Tran	2019	ADRCs	C + AD + MF	AD + MF	auto

QC	1	2	3	4	5	6	7	8
	●	●	●		●	●	●	●

WOMAC Total score reported at 6 months post treatment using bone marrow-derived cells

R	C	T	First author	Y	Cells	Treatment	Control	O
[28]	3	a	Garay-Mendoza	2018	BMACBMAC	C	OA	auto
[53]	4	a	Lamo-Espinosa	2016	BM-MSCs	C + HA	HA	auto
[53]	4	a	Lamo-Espinosa	2016	BM-MSCs	C + HA	HA	auto
[220]			Lv	2015	BM-MSCs	?	?	auto
[14]	1	a	Emadedin	2018	BM-MSCs	C	S	auto

QC	1	2	3	4	5	6	7	8
	●	●	●	●	●	●		

WOMAC Total score reported at 12 months post treatment using bone marrow-derived cells

R	C	T	First author	Y	Cells	Treatment	Control	O
[53]	4	a	Lamo-Espinosa	2016	BM-MSCs	C + HA	HA	auto
[53]	4	a	Lamo-Espinosa	2016	BM-MSCs	C + HA	HA	auto
[220]			Lv	2015	BM-MSCs	?	?	auto
[15]	4	a	Gupta	2016	BM-MSCs	C + HA	S + HA	allo
[15]	4	a	Gupta	2016	BM-MSCs	C + HA	S + HA	allo
[15]	4	a	Gupta	2016	BM-MSCs	C + HA	S + HA	allo
[15]	4	a	Gupta	2016	BM-MSCs	C + HA	S + HA	allo
[20]	2	a	Vega	2015	BM-MSCs	C + HA	HA	allo

QC	1	2	3	4	5	6	7	8
	●	●	●	●			●	

WOMAC Total score reported at 6 months post treatment using adipose-derived cells

R	C	T	First author	Y	Cells	Treatment	Control	O
[16]	1	a	Kuah	2018	ADSCs	C	S	allo
[16]	1	a	Kuah	2018	ADSCs	C	S	allo
[19]	1	a	Garza	2020	ADRCs	C	RS	auto
[19]	1	a	Garza	2020	ADRCs	C	RS	auto
[23]	2	a	Lu	2019	ADSCs	C	HA	auto
[18]	1	a	Lee	2019	ADSCs	C	S	auto

QC	1	2	3	4	5	6	7	8
	●	●	●	●				

WOMAC Total score reported at 12 months post treatment using adipose-derived cells

R	C	T	First author	Y	Cells	Treatment	Control	O
[16]	1	a	Kuah	2018	ADSCs	C	S	allo
[16]	1	a	Kuah	2018	ADSCs	C	S	allo
[19]	1	a	Garza	2020	ADRCs	C	RS	auto
[19]	1	a	Garza	2020	ADRCs	C	RS	auto
[23]	2	a	Lu	2019	ADSCs	C	HA	auto
[30]	3	a	Freitag	2019	ADSCs	C	CM	auto
[30]	3	a	Freitag	2019	ADSCs	C	CM	auto
[69]	4	c	Tran	2019	ADRCs	C + AD + MF	AD + MF	auto

QC	1	2	3	4	5	6	7	8
	●	●	●					

Lysholm score reported at 12 months post treatment using bone marrow-derived cells

R	C	T	First author	Y	Cells	Treatment	Control	O
[218]	6	a	Vangsness	2014	BM-MSCs	C	S	allo
[218]	6	a	Vangsness	2014	BM-MSCs	C	S	allo
[51]	4	a	Wong	2013	BM-MSCs	C + MF + HTO + HA	MF + HTO + HA	auto

QC	1	2	3	4	5	6	7	8
	●		●	●	●		●	

Lysholm score reported at 24 months post treatment using bone marrow-derived cells

R	C	T	First author	Y	Cells	Treatment	Control	O
[218]	6	a	Vangsness	2014	BM-MSCs	C	S	allo
[218]	6	a	Vangsness	2014		C	S	allo
[51]	4	a	Wong	2013	BM-MSCs	C + MF + HTO + HA	MF + HTO + HA	auto

QC	1	2	3	4	5	6	7	8
	●		●	●	●		●	

Lysholm score reported at 24 months post treatment using adipose-derived cells

R	C	T	First author	Y	Cells	Treatment	Control	O
[69]	4	c	Tran	2019	ADRCs	C + AD + MF	AD + MF	auto
[65]	4	c	Koh	2012	ADRCs	C + AD + PRP	AD + PRP	auto
[52]	4	a	Koh	2014	ADRCs	C + AD + HTO + PRP	AD + HTO + PRP	auto

QC	1	2	3	4	5	6	7	8
	●	●	●		●	●	●	●

KOOS score reported at 12 months post treatment using bone marrow-derived cells

R	C	T	First author	Y	Cells	Treatment	Control	O
[32]	3	a	Bastos	2020	BM-MSCs	C	C + PRP	auto
[32]	3	a	Bastos	2020	BM-MSCs	C	C + PRP	auto

QC	1	2	3	4	5	6	7	8
	●	●	●	●	●	●	●	

KOOS score reported at 12 months post treatment using adipose-derived cells

R	C	T	First author	Y	Cells	Treatment	Control	O
[30]	3	a	Freitag	2019	ADSCs	C	CM	auto
[30]	3	a	Freitag	2019	ADSCs	C	CM	auto
[18]	1	a	Lee	2019	ADSCs	C	S	auto

QC	1	2	3	4	5	6	7	8
	●	●	●	●	●	●	●	

WORMS score reported at 12 months post treatment using bone marrow-derived cells

R	C	T	First author	Y	Cells	Treatment	Control	O
[53]	4	a	Lamo-Espinosa	2016	BM-MSCs	C + HA	HA	auto
[53]	4	a	Lamo-Espinosa	2016	BM-MSCs	C + HA	HA	auto
[51]	4	a	Wong	2013	BM-MSCs	C + MF + HTO + HA	MF + HTO + HA	auto
[15]	4	a	Gupta	2016	BM-MSCs	C + HA	S + HA	allo
[15]	4	a	Gupta	2016	BM-MSCs	C + HA	S + HA	allo

QC	1	2	3	4	5	6	7	8
	●	●	●	●	●		●	

WORMS score reported at 12 months post treatment using adipose-derived cells

R	C	T	First author	Y	Cells	Treatment	Control	O
[23]	2	a	Lu	2019	ADSCs	C	HA	auto
[18]	1	a	Lee	2019	ADSCs	C	S	auto
[63]	4	b	Hong	2019	ADRCs	C + AD	HA + AD	auto

QC	1	2	3	4	5	6	7	8
	●	●	●	0	0	●	0	●

Adverse events reported after treating pkOA using bone marrow-derived cells

R	C	T	First author	Y	Cells	Treatment	Control	O
[28]	3	a	Garay-Mendoza	2018	BMACBMAC	C	OA	auto
[53]	4	a	Lamo-Espinosa	2016	BM-MSCs	C + HA	HA	auto
[53]	4	a	Lamo-Espinosa	2016	BM-MSCs	C + HA	HA	auto

QC	1	2	3	4	5	6	7	8
	●	●	●	●	●	●	●	

Adverse events reported after treating pkOA using adipose-derived cells

R	C	T	First author	Y	Cells	Treatment	Control	O
[16]	1	a	Kuah	2018	ADSCs	C	S	allo
[16]	1	a	Kuah	2018	ADSCs	C	S	allo
[18]	1	a	Lee	2019	ADSCs	C	S	auto

QC	1	2	3	4	5	6	7	8
	●	●	●	●	●		●	●

Table S11 | Details of the sub-analyses performed in a meta-analysis by Han et al. [209].

Abbreviations: R, reference number; C, category of study as outlined in Table 2 in the main text; T, type of study as outlined in Table 3 in the main text; Y, year of publication; O, origin of cells (allo, allogeneic cells; auto; autologous cells); QC, quality criteria (outlined in Table 6 in the main text; a point indicates that the corresponding quality criterion was fulfilled). The abbreviations of the cell types and treatments are provided in Table S1.

VAS Pain score M6 bone marrow-derived cells

R	C	T	First author	Y	Cells	Treatment	Control	O
[53]	4	a	Lamo-Espinosa	2016	BM-MSCs	C + HA	HA	auto
[53]	4	a	Lamo-Espinosa	2016	BM-MSCs	C + HA	HA	auto
[218]	6	a	Vangsness	2014	BM-MSCs	C	S	allo
[218]	6	a	Vangsness	2014	BM-MSCs	C	S	allo

QC	1	2	3	4	5	6	7	8
	●		●	●	●	●	●	

VAS Pain score M6 adipose-derived cells

R	C	T	First author	Y	Cells	Treatment	Control	O
[23]	2	a	Lu	2019	ADSCs	C	HA	auto
[23]	2	a	Lu	2019	ADSCs	C	HA	auto

QC	1	2	3	4	5	6	7	8
	●	●	●	●		●	●	

VAS Pain score M12 bone marrow-derived cells

R	C	T	First author	Y	Cells	Treatment	Control	O
[15]	1	a	Gupta	2016	BM-MSCs	C	S	allo
[15]	1	a	Gupta	2016	BM-MSCs	C	S	allo
[53]	4	a	Lamo-Espinosa	2016	BM-MSCs	C + HA	HA	auto
[53]	4	a	Lamo-Espinosa	2016	BM-MSCs	C + HA	HA	auto
[218]	6	a	Vangsness	2014	BM-MSCs	C	S	allo
[218]	6	a	Vangsness	2014	BM-MSCs	C	S	allo
[20]	2	a	Vega	2015	BM-MSCs	C	HA	allo

QC	1	2	3	4	5	6	7	8
	●		●	●	●		●	

VAS Pain score M12 adipose-derived cells

R	C	T	First author	Y	Cells	Treatment	Control	O
[65]	4	c	Koh	2012	ADSCs	C + AD + PRP	AD + PRP	auto
[23]	2	a	Lu	2019	ADSCs	C	HA	auto
[23]	2	a	Lu	2019	ADSCs	C	HA	auto

QC	1	2	3	4	5	6	7	8
	●	●	●			●	●	

VAS Pain score M24 bone marrow-derived cells

R	C	T	First author	Y	Cells	Treatment	Control	O
[218]	6	a	Vangsness	2014	BM-MSCs	C	S	allo
[218]	6	a	Vangsness	2014	BM-MSCs	C	S	allo

QC	1	2	3	4	5	6	7	8
	●		●	●	●	●	●	

VAS Pain score M24 adipose-derived cells

R	C	T	First author	Y	Cells	Treatment	Control	O
[65]	4	c	Koh	2012	ADSCs	C + AD + PRP	AD + PRP	auto

QC	1	2	3	4	5	6	7	8
		●	●		●	●	●	●

WOMAC Total score at 6 months post treatment using bone marrow-derived cells

R	C	T	First author	Y	Cells	Treatment	Control	O
[53]	4	a	Lamo-Espinosa	2016	BM-MSCs	C + HA	HA	auto
[53]	4	a	Lamo-Espinosa	2016	BM-MSCs	C + HA	HA	auto

QC	1	2	3	4	5	6	7	8
		●	●	●	●	●	●	

WOMAC Total score at 6 months post treatment using adipose-derived cells

R	C	T	First author	Y	Cells	Treatment	Control	O
[30]	3	a	Freitag	2019	ADSCs	C	CM	auto
[30]	3	a	Freitag	2019	ADSCs	C	CM	auto
[23]	2	a	Lu	2019	ADSCs	C	HA	auto

QC	1	2	3	4	5	6	7	8
	●	●	●	●		●	●	

WOMAC Total score at 12 months post treatment using bone marrow-derived cells

R	C	T	First author	Y	Cells	Treatment	Control	O
[15]	4	a	Gupta	2016	BM-MSCs	C + HA	S + HA	allo
[15]	4	a	Gupta	2016	BM-MSCs	C + HA	S + HA	allo
[53]	4	a	Lamo-Espinosa	2016	BM-MSCs	C + HA	HA	auto
[53]	4	a	Lamo-Espinosa	2016	BM-MSCs	C + HA	HA	auto
[20]	2	a	Vega	2015	BM-MSCs	C	HA	allo

QC	1	2	3	4	5	6	7	8
	●	●	●	●				

WOMAC Total score at 12 months post treatment using adipose-derived cells

R	C	T	First author	Y	Cells	Treatment	Control	O
[30]	3	a	Freitag	2019	ADSCs	C	CM	auto
[30]	3	a	Freitag	2019	ADSCs	C	CM	auto
[23]	2	a	Lu	2019	ADSCs	C	HA	auto

QC	1	2	3	4	5	6	7	8
	●	●	●	●		●	●	

Lysholm score at 12 months post treatment using bone marrow-derived cells

R	C	T	First author	Y	Cells	Treatment	Control	O
[218]	6	a	Vangsness	2014	BM-MSCs	C	S	allo
[218]	6	a	Vangsness	2014	BM-MSCs	C	S	allo
[51]	4	a	Wong	2013	BM-MSCs	C + MF + HTO + HA	MF + HTO + HA	auto

QC	1	2	3	4	5	6	7	8
●		●	●	●	●			●

Lysholm score at 12 months post treatment using adipose-derived cells

R	C	T	First author	Y	Cells	Treatment	Control	O
[65]	4	c	Koh	2012	ADSCs	C + AD + PRP	AD + PRP	auto

QC	1	2	3	4	5	6	7	8
		●	●		●	●	●	●

Lysholm score at 24 months post treatment using bone marrow-derived cells

R	C	T	First author	Y	Cells	Treatment	Control	O
[218]	6	a	Vangsness	2014	BM-MSCs	C	S	allo
[218]	6	a	Vangsness	2014	BM-MSCs	C	S	allo
[51]	4	a	Wong	2013	BM-MSCs	C + MF + HTO + HA	MF + HTO + HA	auto

QC	1	2	3	4	5	6	7	8
	●		●	●	●	●		

Lysholm score at 24 months post treatment using adipose-derived cells

R	C	T	First author	Y	Cells	Treatment	Control	O
[65]	4	c	Koh	2012	ADSCs	C + AD + PRP	AD + PRP	auto

QC	1	2	3	4	5	6	7	8
		●	●		●	●	●	●

Tegner score at 12 months post treatment using bone marrow-derived cells

R	C	T	First author	Y	Cells	Treatment	Control	O
[51]	4	a	Wong	2013	BM-MSCs	C + MF + HTO + HA	MF + HTO + HA	auto

QC	1	2	3	4	5	6	7	8
		●	●	●	●	●	●	●

Tegner score at 12 months post treatment using adipose-derived cells

R	C	T	First author	Y	Cells	Treatment	Control	O
[65]	4	c	Koh	2012	ADSCs	C + AD + PRP	AD + PRP	auto

QC	1	2	3	4	5	6	7	8
		●	●		●	●	●	●

Table S12 | Details of the sub-analyses performed in a meta-analysis by Kim et al. [213].

Abbreviations: R, reference number; C, category of study as outlined in Table 2 in the main text; T, type of study as outlined in Table 3 in the main text; Y, year of publication; O, origin of cells (allo, allogeneic cells; auto; autologous cells); QC, quality criteria (outlined in Table 6 in the main text; a point indicates that the corresponding quality criterion was fulfilled). The abbreviations of the cell types and treatments are provided in Table S1.

VAS Pain score

R	C	T	First author	Y	Cells	Treatment	Control	O
[20]	2	a	Vega	2015	BM-MSCs	C	HA	allo
[53]	4	a	Lamo-Espinosa	2016	BM-MSCs	C + HA	HA	auto
[15]	4	a	Gupta	2016	BM-MSCs	C + HA	S + HA	allo
[16]	1	a	Kuah	2018	ADSCs	C	S	allo
[14]	1	a	Emadedin	2018	BM-MSCs	C	S	auto

QC	1	2	3	4	5	6	7	8
	●	●	●	●			●	●

WOMAC Total score

R	C	T	First author	Y	Cells	Treatment	Control	O
[20]	2	a	Vega	2015	BM-MSCs	C	HA	allo
[15]	4	a	Gupta	2016	BM-MSCs	C + HA	S + HA	allo
[53]	4	a	Lamo-Espinosa	2016	BM-MSCs	C + HA	HA	auto
[14]	1	a	Emadedin	2018	BM-MSCs	C	S	auto

QC	1	2	3	4	5	6	7	8
	●	●	●	●			●	●

WOMAC Pain score

R	C	T	First author	Y	Cells	Treatment	Control	O
[14]	1	a	Emadedin	2018	BM-MSCs	C	S	auto
[15]	4	a	Gupta	2016	BM-MSCs	C + HA	S + HA	allo
[16]	1	a	Kuah	2018	ADSCs	C	S	allo
[53]	4	a	Lamo-Espinosa	2016	BM-MSCs	C + HA	HA	auto
[20]	2	a	Vega	2015	BM-MSCs	C	HA	allo

QC	1	2	3	4	5	6	7	8
	●	●	●	●			●	●

WOMAC Function score

R	C	T	First author	Y	Cells	Treatment	Control	O
[14]	1	a	Emadedin	2018	BM-MSCs	C	S	auto
[15]	4	a	Gupta	2016	BM-MSCs	C + HA	S + HA	allo
[53]	4	a	Lamo-Espinosa	2016	BM-MSCs	C + HA	HA	auto

QC	1	2	3	4	5	6	7	8
	●	●	●	●	●		●	●

WORMS score

R	C	T	First author	Y	Cells	Treatment	Control	O
[53]	4	a	Lamo-Espinosa	2016	BM-MSCs	C + HA	HA	auto
[15]	4	a	Gupta	2016	BM-MSCs	C + HA	S + HA	allo

QC	1	2	3	4	5	6	7	8
	●	●	●	●	●		●	●

Categorical evaluations of MRI data

R	C	T	First author	Y	Cells	Treatment	Control	O
[17]	1	a	Khalifeh Soltani	2019	P-MSCs	C	S	allo
[16]	1	a	Kuah	2018	ADSCs	C	S	allo

QC	1	2	3	4	5	6	7	8
	●	●	●	●	●	●	●	●

Cumulative pain scores (WOMAC and VAS): WOMAC

R	C	T	First author	Y	Cells	Treatment	Control	O
[20]	2	a	Vega	2015	BM-MSCs	C	HA	allo
[15]	4	a	Gupta	2016	BM-MSCs	C + HA	S + HA	allo
[53]	4	a	Lamo-Espinosa	2016	BM-MSCs	C + HA	HA	auto
[16]	1	a	Kuah	2018	ADSCs	C	S	allo
[14]	1	a	Emadedin	2018	BM-MSCs	C	S	auto

QC	1	2	3	4	5	6	7	8
	●	●	●	●			●	●

Cumulative pain scores (WOMAC and VAS): VAS

R	C	T	First author	Y	Cells	Treatment	Control	O
[20]	2	a	Vega	2015	BM-MSCs	C	HA	allo
[15]	4	a	Gupta	2016	BM-MSCs	C + HA	S + HA	allo
[53]	4	a	Lamo-Espinosa	2016	BM-MSCs	C + HA	HA	auto
[14]	1	a	Emadedin	2018	BM-MSCs	C	S	auto
[16]	1	a	Kuah	2018	ADSCs	C	S	allo

QC	1	2	3	4	5	6	7	8
	●	●	●	●			●	●

Table S13 | Details of the sub-analyses performed in a meta-analysis by Ding et al. [214].

Abbreviations: R, reference number; C, category of study as outlined in Table 2 in the main text; T, type of study as outlined in Table 3 in the main text; Y, year of publication; O, origin of cells (allo, allogeneic cells; auto; autologous cells); QC, quality criteria (outlined in Table 6 in the main text; a point indicates that the corresponding quality criterion was fulfilled). The abbreviations of the cell types and treatments are provided in Table S1.

WOMAC Total score reported at 6 months post treatment using allogeneic cells

R	C	T	First author	Y	Cells	Treatment	Control	O
[24]	2	a	Matas	2019	hUC-MSCs	C	HA	allo
[24]	2	a	Matas	2019	hUC-MSCs	C	HA	allo
[16]	1	a	Kuah	2018	ADSCs	C	S	allo
[16]	1	a	Kuah	2018	ADSCs	C	S	allo
[21]	2	a	Wang	2016	hUC-MSCs	C	HA	allo
[15]	4	a	Gupta	2016	BM-MSCs	C + HA	S + HA	allo
[15]	4	a	Gupta	2016	BM-MSCs	C + HA	S + HA	allo
[15]	4	a	Gupta	2016	BM-MSCs	C + HA	S + HA	allo
[15]	4	a	Gupta	2016	BM-MSCs	C + HA	S + HA	allo
[20]	2	a	Vega	2015	BM-MSCs	C	HA	allo

QC	1	2	3	4	5	6	7	8
	●	●	●	●		●	●	

WOMAC Total score reported at 6 months post treatment using autologous cells

R	C	T	First author	Y	Cells	Treatment	Control	O
[23]	2	a	Lu	2019	ADSCs	C	HA	auto
[30]	3	a	Freitag	2019	ADSCs	C	CM	auto
[30]	3	a	Freitag	2019	ADSCs	C	CM	auto
[53]	4	a	Lamo-Espinosa	2016	BM-MSCs	C + HA	HA	auto
[53]	4	a	Lamo-Espinosa	2016	BM-MSCs	C + HA	HA	auto
[14]	1	a	Emadedin	2018	BM-MSCs	C	S	auto

QC	1	2	3	4	5	6	7	8
	●	●	●	●		●	●	

KOOS score reported at 12 months post treatment using allogeneic cells

R	C	T	First author	Y	Cells	Treatment	Control	O
[15]	1	a	Kim	2018	Ch-TGFß	C	S	allo

QC	1	2	3	4	5	6	7	8
		●	●	●	●	●	●	●

KOOS score reported at 12 months post treatment using autologous cells

R	C	T	First author	Y	Cells	Treatment	Control	O
[30]	3	a	Freitag	2019	ADSCs	C	CM	auto
[30]	3	a	Freitag	2019	ADSCs	C	CM	auto
[55]	4	a	Bastos	2018	BM-MSCs	C + PRP	C	auto
[55]	4	a	Bastos	2018	BM-MSCs	C + PRP	C	auto

QC	1	2	3	4	5	6	7	8
	●	●	●	●	●	●	●	

VAS Pain score reported at 6 months post treatment using allogeneic cells

R	C	T	First author	Y	Cells	Treatment	Control	O
[24]	2	a	Matas	2019	hUC-MSCs	C	HA	allo
[24]	2	a	Matas	2019	hUC-MSCs	C	HA	allo
[16]	1	a	Kuah	2018	ADSCs	C	S	allo
[16]	1	a	Kuah	2018	ADSCs	C	S	allo
[15]	1	a	Kim	2018	Ch-TGFß	C	S	allo
[15]	4	a	Gupta	2016	BM-MSCs	C + HA	S + HA	allo
[15]	4	a	Gupta	2016	BM-MSCs	C + HA	S + HA	allo
[15]	4	a	Gupta	2016	BM-MSCs	C + HA	S + HA	allo
[15]	4	a	Gupta	2016	BM-MSCs	C + HA	S + HA	allo
[20]	2	a	Vega	2015	BM-MSCs	C	HA	allo

QC	1	2	3	4	5	6	7	8
	●	●	●	●		●	●	

VAS Pain score reported at 6 months post treatment using autologous cells

R	C	T	First author	Y	Cells	Treatment	Control	O
[23]	2	a	Lu	2019	ADSCs	C	HA	auto
[30]	3	a	Freitag	2019	ADSCs	C	CM	auto
[30]	3	a	Freitag	2019	ADSCs	C	CM	auto
[53]	4	a	Lamo-Espinosa	2016	BM-MSCs	C + HA	HA	auto
[53]	4	a	Lamo-Espinosa	2016	BM-MSCs	C + HA	HA	auto
[14]	1	a	Emadedin	2018	BM-MSCs	C	S	auto

QC	1	2	3	4	5	6	7	8
	●	●	●	●		●	●	

Adverse events reported after treating pkOA using allogeneic cells

R	C	T	First author	Y	Cells	Treatment	Control	O
[24]	2	a	Matas	2019	hUC-MSCs	C	HA	allo
[24]	2	a	Matas	2019	hUC-MSCs	C	HA	allo
[16]	1	a	Kuah	2018	ADSCs	C	S	allo
[16]	1	a	Kuah	2018	ADSCs	C	S	allo
[15]	1	a	Kim	2018	Ch-TGFß	C	S	allo
[21]	2	a	Wang	2016	hUC-MSCs	C	HA	allo
[15]	4	a	Gupta	2016	BM-MSCs	C + HA	S + HA	allo
[15]	4	a	Gupta	2016	BM-MSCs	C + HA	S + HA	allo
[15]	4	a	Gupta	2016	BM-MSCs	C + HA	S + HA	allo
[15]	4	a	Gupta	2016	BM-MSCs	C + HA	S + HA	allo
[20]	2	a	Vega	2015	BM-MSCs	C	HA	allo
[12]	1	a	Cherian	2015	Ch-TGFß	C	S	allo

QC	1	2	3	4	5	6	7	8
	●	●	●	●		●	●	

Adverse events reported after treating pkOA using autologous cells

R	C	T	First author	Y	Cells	Treatment	Control	O
[23]	2	a	Lu	2019	ADSCs	C	HA	auto
[18]	1	a	Lee	2019	ADSCs	C	S	auto
[30]	3	a	Freitag	2019	ADSCs	C	CM	auto
[30]	3	a	Freitag	2019	ADSCs	C	CM	auto
[14]	1	a	Emadedin	2018	BM-MSCs	C	S	auto

QC	1	2	3	4	5	6	7	8
	●	●	●	●		●	●	●

Table S14 | Details of the sub-analyses performed in a meta-analysis by Maheshwer et al. [215].

Abbreviations: R, reference number; C, category of study as outlined in Table 2 in the main text; T, type of study as outlined in Table 3 in the main text; Y, year of publication; O, origin of cells (allo, allogeneic cells; auto; autologous cells); QC, quality criteria (outlined in Table 6 in the main text; a point indicates that the corresponding quality criterion was fulfilled). The abbreviations of the cell types and treatments are provided in Table S1.

VAS Pain score

R	C	T	First author	Y	Cells	Treatment	Control	O
[65]	4	c	Koh	2012	ADSCs	C + AD + PRP	AD + PRP	auto
[52]	4	a	Koh	2014	ADRCs	C + AD + HTO + PRP	AD + HTO + PRP	auto
[157]	6	a	Akgun	2015	S-MSCs	C in S + AD	MACI	auto
[20]	2	a	Vega	2015	BM-MSCs	C	HA	allo
[15]	4	a	Gupta	2016	BM-MSCs	C + HA	S + HA	allo
[15]	4	a	Gupta	2016	BM-MSCs	C + HA	S + HA	allo
[15]	4	a	Gupta	2016	BM-MSCs	C + HA	S + HA	allo
[15]	4	a	Gupta	2016	BM-MSCs	C + HA	S + HA	allo
[23]	2	a	Lu	2019	ADSCs	C	HA	auto
[23]	2	a	Lu	2019	ADSCs	C	HA	auto

QC	1	2	3	4	5	6	7	8
	●	●	●					

VAS Pain score after treating pkOA using stem cells

R	C	T	First author	Y	Cells	Treatment	Control	O
[20]	2	a	Vega	2015	BM-MSCs	C	HA	allo
[15]	4	a	Gupta	2016	BM-MSCs	C + HA	S + HA	allo
[15]	4	a	Gupta	2016	BM-MSCs	C + HA	S + HA	allo
[15]	4	a	Gupta	2016	BM-MSCs	C + HA	S + HA	allo
[15]	4	a	Gupta	2016	BM-MSCs	C + HA	S + HA	allo
[23]	2	a	Lu	2019	ADSCs	C	HA	auto
[23]	2	a	Lu	2019	ADSCs	C	HA	auto

QC	1	2	3	4	5	6	7	8
	●	●	●	●			●	

VAS Pain score after treating pkOA with stem cells in conjunction with surgery

R	C	T	First author	Y	Cells	Treatment	Control	O
[65]	4	c	Koh	2012	ADSCs	C + AD + PRP	AD + PRP	auto
[52]	4	a	Koh	2014	ADRCs	C + AD + HTO + PRP	AD + HTO + PRP	auto
[157]	6	a	Akgun	2015	S-MSCs	C in S + AD	MACI	auto

QC	1	2	3	4	5	6	7	8
	●	●	●			●	●	●

WOMAC Total score

R	C	T	First author	Y	Cells	Treatment	Control	O
[20]	2	a	Vega	2015	BM-MSCs	C	HA	allo
[15]	4	a	Gupta	2016	BM-MSCs	C + HA	S + HA	allo
[15]	4	a	Gupta	2016	BM-MSCs	C + HA	S + HA	allo

QC	1	2	3	4	5	6	7	8
	●	●	●	●		●	●	

IKDC scoore

R	C	T	First author	Y	Cells	Treatment	Control	O
[15]	4	c	Kim	2018	ADRCs	C + AD + HTO	AD + HTO	auto

QC	1	2	3	4	5	6	7	8
		●	●		●	●	●	●

Lysholm score

R	C	T	First author	Y	Cells	Treatment	Control	O
[65]	4	c	Koh	2012	ADSCs	C + AD + PRP	AD + PRP	auto
[52]	4	a	Koh	2014	ADRCs	C + AD + HTO + PRP	AD + HTO + PRP	auto

QC	1	2	3	4	5	6	7	8
	●	●	●		●	●		●

KOOS score

R	C	T	First author	Y	Cells	Treatment	Control	O
[157]	6	a	Akgun	2015	S-MSCs	C in S + AD	MACI	auto

QC	1	2	3	4	5	6	7	8
		●	●	●		●	●	●

Physical function after treating pkOA using stem cells

R	C	T	First author	Y	Cells	Treatment	Control	O
[20]	2	a	Vega	2015	BM-MSCs	C	HA	allo
[15]	4	a	Gupta	2016	BM-MSCs	C + HA	S + HA	allo
[15]	4	a	Gupta	2016	BM-MSCs	C + HA	S + HA	allo
[23]	2	a	Lu	2019	ADSCs	C	HA	auto

QC	1	2	3	4	5	6	7	8
	●	●	●	●			●	

Physical function after treating pkOA with stem cells in conjunction with surgery

R	C	T	First author	Y	Cells	Treatment	Control	O
[65]	4	c	Koh	2012	ADSCs	C + AD + PRP	AD + PRP	auto
[157]	6	a	Akgun	2015	S-MSCs	C in S + AD	MACI	auto
[15]	4	c	Kim	2018	ADRCs	C + AD + HTO	AD + HTO	auto
[52]	4	a	Koh	2014	ADRCs	C + AD + HTO + PRP	AD + HTO + PRP	auto

QC	1	2	3	4	5	6	7	8
	●	●	●			●		●

Cartilage volume

R	C	T	First author	Y	Cells	Treatment	Control	O
[18]	1	a	Lee	2019	ADSCs	C	S	auto
[23]	2	a	Lu	2019	ADSCs	C	HA	auto
[23]	2	a	Lu	2019	ADSCs	C	HA	auto
[23]	2	a	Lu	2019	ADSCs	C	HA	auto
[23]	2	a	Lu	2019	ADSCs	C	HA	auto

QC	1	2	3	4	5	6	7	8
	●	●	●	●		●	●	

MOCART index (cartilage quality)

R	C	T	First author	Y	Cells	Treatment	Control	O
[161]	6	a	Hashimoto	2019	BM-MSCs	C + MF	MF	auto

QC	1	2	3	4	5	6	7	8
		●	●	●	●	●	●	●

Poor cartilage index

R	C	T	First author	Y	Cells	Treatment	Control	O
[20]	2	a	Vega	2015	BM-MSCs	C	HA	allo

QC	1	2	3	4	5	6	7	8
		●	●	●		●	●	●

WORMS score

R	C	T	First author	Y	Cells	Treatment	Control	O
[15]	4	a	Gupta	2016	BM-MSCs	C	S	allo
[15]	4	a	Gupta	2016	BM-MSCs	C + HA	S + HA	allo
[15]	4	a	Gupta	2016	BM-MSCs	C + HA	S + HA	allo
[15]	4	a	Gupta	2016	BM-MSCs	C + HA	S + HA	allo

QC	1	2	3	4	5	6	7	8
	●	●	●	●	●	●	●	

Table S15 | Details of the sub-analyses performed in a meta-analysis by Dai et al. [216].

Abbreviations: R, reference number; C, category of study as outlined in Table 2 in the main text; T, type of study as outlined in Table 3 in the main text; Y, year of publication; O, origin of cells (allo, allogeneic cells; auto; autologous cells); QC, quality criteria (outlined in Table 6 in the main text; a point indicates that the corresponding quality criterion was fulfilled). The abbreviations of the cell types and treatments are provided in Table S1.

VAS Pain score MSCs vs control

R	C	T	First author	Y	Cells	Treatment	Control	O
[14]	1	a	Emadedin	2018	BM-MSCs	C	S	auto
[15]	1	a	Gupta	2016	BM-MSCs	C	S	allo
[63]	4	b	Hong	2019	ADRCs	C + AD	HA + AD	auto
[16]	1	a	Kuah	2018	ADSCs	C	S	allo
[53]	4	a	Lamo-Espinosa	2016	BM-MSCs	C + HA	HA	auto
[23]	2	a	Lu	2019	ADSCs	C	HA	auto
[24]	2	a	Matas	2019	hUC-MSCs	C	HA	allo
[64]	4	b	Shapiro	2019	BMACBMAC	C + PPP	S	auto
[20]	2	a	Vega	2015	BM-MSCs	C	HA	allo

QC	1	2	3	4	5	6	7	8
	●	●	●					●

VAS Pain score MSC vs placebo

R	C	T	First author	Y	Cells	Treatment	Control	O
[14]	1	a	Emadedin	2018	BM-MSCs	C	S	auto
[16]	1	a	Kuah	2018	ADSCs	C	S	allo
[53]	4	a	Lamo-Espinosa	2016	BM-MSCs	C + HA	HA	auto
[64]	4	b	Shapiro	2019	BMACBMAC	C + PPP	S	auto

QC	1	2	3	4	5	6	7	8
	●	●	●					●

VAS Pain score MSC vs HA

R	C	T	First author	Y	Cells	Treatment	Control	O
[15]	1	a	Gupta	2016	BM-MSCs	C	S	allo
[63]	4	b	Hong	2019	ADRCs	C + AD	HA + AD	auto
[23]	2	a	Lu	2019	ADSCs	C	HA	auto
[24]	2	a	Matas	2019	hUC-MSCs	C	HA	allo
[20]	2	a	Vega	2015	BM-MSCs	C	HA	allo

QC	1	2	3	4	5	6	7	8
	●	●	●					●

WOMAC Total score MSCs vs control

R	C	T	First author	Y	Cells	Treatment	Control	O
[14]	1	a	Emadedin	2018	BM-MSCs	C	S	auto
[30]	3	a	Freitag	2019	ADSCs	C	CM	auto
[19]	1	a	Garza	2020	ADRCs	C	RS	auto
[15]	1	a	Gupta	2016	BM-MSCs	C	S	allo
[53]	4	a	Lamo-Espinosa	2016	BM-MSCs	C + HA	HA	auto
[23]	2	a	Lu	2019	ADSCs	C	HA	auto
[24]	2	a	Matas	2019	hUC-MSCs	C	HA	allo
[20]	2	a	Vega	2015	BM-MSCs	C	HA	allo

QC	1	2	3	4	5	6	7	8
	●	●	●	●				●

WOMAC Total score MSC vs placebo

R	C	T	First author	Y	Cells	Treatment	Control	O
[14]	1	a	Emadedin	2018	BM-MSCs	C	S	auto
[30]	3	a	Freitag	2019	ADSCs	C	CM	auto
[19]	1	a	Garza	2020	ADRCs	C	RS	auto
[53]	4	a	Lamo-Espinosa	2016	BM-MSCs	C + HA	HA	auto

QC	1	2	3	4	5	6	7	8
	●	●	●	●	●	●		●

WOMAC Total score MSC vs HA

R	C	T	First author	Y	Cells	Treatment	Control	O
[15]	1	a	Gupta	2016	BM-MSCs	C	S	allo
[23]	2	a	Lu	2019	ADSCs	C	HA	auto
[24]	2	a	Matas	2019	hUC-MSCs	C	HA	allo
[20]	2	a	Vega	2015	BM-MSCs	C	HA	allo

QC	1	2	3	4	5	6	7	8
	●	●	●	●			●	●

VAS Pain score Adipose-derived MSCs

R	C	T	First author	Y	Cells	Treatment	Control	O
[63]	4	b	Hong	2019	ADRCs	C + AD	HA + AD	auto
[16]	1	a	Kuah	2018	ADSCs	C	S	allo
[23]	2	a	Lu	2019	ADSCs	C	HA	auto

QC	1	2	3	4	5	6	7	8
	●	●	●					●

VAS Pain score Bone marrow-derived MSCs

R	C	T	First author	Y	Cells	Treatment	Control	O
[14]	1	a	Emadedin	2018	BM-MSCs	C	S	auto
[15]	1	a	Gupta	2016	BM-MSCs	C	S	allo
[53]	4	a	Lamo-Espinosa	2016	BM-MSCs	C + HA	HA	auto
[64]	4	b	Shapiro	2019	BMACBMAC	C + PPP	S	auto
[20]	2	a	Vega	2015	BM-MSCs	C	HA	allo

QC	1	2	3	4	5	6	7	8
	●	●	●					●

VAS Pain score Umbilical cord-derived MSCs

R	C	T	First author	Y	Cells	Treatment	Control	O
[24]	2	a	Matas	2019	hUC-MSCs	C	HA	allo

QC	1	2	3	4	5	6	7	8
		●	●	●		●	●	●

WOMAC Total score Adipose-derived MSCs

R	C	T	First author	Y	Cells	Treatment	Control	O
[30]	3	a	Freitag	2019	ADSCs	C	CM	auto
[19]	1	a	Garza	2020	ADRCs	C	RS	auto
[23]	2	a	Lu	2019	ADSCs	C	HA	auto

QC	1	2	3	4	5	6	7	8
	●	●	●	●		●		●

WOMAC Total score Bone marrow-derived MSCs

R	C	T	First author	Y	Cells	Treatment	Control	O
[14]	1	a	Emadedin	2018	BM-MSCs	C	S	auto
[15]	1	a	Gupta	2016	BM-MSCs	C	S	allo
[53]	4	a	Lamo-Espinosa	2016	BM-MSCs	C + HA	HA	auto
[20]	2	a	Vega	2015	BM-MSCs	C	HA	allo

QC	1	2	3	4	5	6	7	8
	●	●	●	●			●	●

WOMAC Total score Umbilical cord-derived MSCs

R	C	T	First author	Y	Cells	Treatment	Control	O
[24]	2	a	Matas	2019	hUC-MSCs	C	HA	allo

QC	1	2	3	4	5	6	7	8
		●	●	●		●	●	●

VAS Pain score Cultured cells

R	C	T	First author	Y	Cells	Treatment	Control	O
[14]	1	a	Emadedin	2018	BM-MSCs	C	S	auto
[15]	1	a	Gupta	2016	BM-MSCs	C	S	allo
[16]	1	a	Kuah	2018	ADSCs	C	S	allo
[53]	4	a	Lamo-Espinosa	2016	BM-MSCs	C + HA	HA	auto
[23]	2	a	Lu	2019	ADSCs	C	HA	auto
[24]	2	a	Matas	2019	hUC-MSCs	C	HA	allo
[20]	2	a	Vega	2015	BM-MSCs	C	HA	allo

QC	1	2	3	4	5	6	7	8
	●	●	●	●			●	●

VAS Pain score Uncultured cells

R	C	T	First author	Y	Cells	Treatment	Control	O
[63]	4	b	Hong	2019	ADRCs	C + AD	HA + AD	auto
[64]	4	b	Shapiro	2019	BMACBMAC	C + PPP	S	auto

QC	1	2	3	4	5	6	7	8
	●	●	●			●	●	●

WOMAC Total score Cultured cells

R	C	T	First author	Y	Cells	Treatment	Control	O
[14]	1	a	Emadedin	2018	BM-MSCs	C	S	auto
[30]	3	a	Freitag	2019	ADSCs	C	CM	auto
[15]	1	a	Gupta	2016	BM-MSCs	C	S	allo
[53]	4	a	Lamo-Espinosa	2016	BM-MSCs	C + HA	HA	auto
[23]	2	a	Lu	2019	ADSCs	C	HA	auto
[24]	2	a	Matas	2019	hUC-MSCs	C	HA	allo
[20]	2	a	Vega	2015	BM-MSCs	C	HA	allo

QC	1	2	3	4	5	6	7	8
	●	●	●	●			●	●

WOMAC Total score Uncultured cells

R	C	T	First author	Y	Cells	Treatment	Control	O
[19]	1	a	Garza	2020	ADRCs	C	RS	auto

QC	1	2	3	4	5	6	7	8
		●	●	●	●	●	●	●

VAS Pain score Autologous cells

R	C	T	First author	Y	Cells	Treatment	Control	O
[14]	1	a	Emadedin	2018	BM-MSCs	C	S	auto
[63]	4	b	Hong	2019	ADRCs	C + AD	HA + AD	auto
[53]	4	a	Lamo-Espinosa	2016	BM-MSCs	C + HA	HA	auto
[23]	2	a	Lu	2019	ADSCs	C	HA	auto
[64]	4	b	Shapiro	2019	BMACBMAC	C + PPP	S	auto

QC	1	2	3	4	5	6	7	8
	●	●	●			●		●

VAS Pain score Allogeneic cells

R	C	T	First author	Y	Cells	Treatment	Control	O
[15]	1	a	Gupta	2016	BM-MSCs	C	S	allo
[16]	1	a	Kuah	2018	ADSCs	C	S	allo
[24]	2	a	Matas	2019	hUC-MSCs	C	HA	allo
[20]	2	a	Vega	2015	BM-MSCs	C	HA	allo

QC	1	2	3	4	5	6	7	8
	●	●	●	●		●	●	●

WOMAC Total score Autologous cells

R	C	T	First author	Y	Cells	Treatment	Control	O
[14]	1	a	Emadedin	2018	BM-MSCs	C	S	auto
[30]	3	a	Freitag	2019	ADSCs	C	CM	auto
[19]	1	a	Garza	2020	ADRCs	C	RS	auto
[53]	4	a	Lamo-Espinosa	2016	BM-MSCs	C + HA	HA	auto
[23]	2	a	Lu	2019	ADSCs	C	HA	auto

QC	1	2	3	4	5	6	7	8
	●	●	●	●		●		●

WOMAC Total score Allogeneic cells

R	C	T	First author	Y	Cells	Treatment	Control	O
[15]	1	a	Gupta	2016	BM-MSCs	C	S	allo
[24]	2	a	Matas	2019	hUC-MSCs	C	HA	allo
[21]	2	a	Vega	2015	BM-MSCs	C	HA	allo

QC	1	2	3	4	5	6	7	8
	●	●	●	●		●	●	●
